# Drug-regulated CD33-targeted CAR T cells control AML using clinically optimized rapamycin dosing

**DOI:** 10.1172/JCI162593

**Published:** 2024-03-19

**Authors:** Jacob Appelbaum, April E. Price, Kaori Oda, Joy Zhang, Wai-Hang Leung, Giacomo Tampella, Dong Xia, Pauline P.L. So, Sarah K. Hilton, Claudya Evandy, Semanti Sarkar, Unja Martin, Anne-Rachel Krostag, Marissa Leonardi, Daniel E. Zak, Rachael Logan, Paula Lewis, Secil Franke-Welch, Njabulo Ngwenyama, Michael Fitzgerald, Niklas Tulberg, Stephanie Rawlings-Rhea, Rebecca A. Gardner, Kyle Jones, Angelica Sanabria, William Crago, John Timmer, Andrew Hollands, Brendan Eckelman, Sanela Bilic, Jim Woodworth, Adam Lamble, Philip D. Gregory, Jordan Jarjour, Mark Pogson, Joshua A. Gustafson, Alexander Astrakhan, Michael C. Jensen

**Affiliations:** 1 Seattle Children’s Therapeutics, Seattle Children’s Research Institute, Seattle, Washington, USA.; 2 Division of Hematology/Oncology, Department of Medicine, University of Washington School of Medicine, Seattle, Washington, USA.; 3 Fred Hutchinson Cancer Center, Seattle, Washington, USA.; 4 Seattle Children’s Hospital, Seattle, Washington, USA.; 5 2seventy bio, Cambridge, Massachusetts, USA.; 6 Inhibrx, Torrey Pines Science Park, La Jolla, California, USA.; 7 Vandro Consulting, Waukee, Iowa, USA.

**Keywords:** Hematology, Therapeutics, Cancer immunotherapy, Leukemias, T cells

## Abstract

Chimeric antigen receptor (CAR) designs that incorporate pharmacologic control are desirable; however, designs suitable for clinical translation are needed. We designed a fully human, rapamycin-regulated drug product for targeting CD33^+^ tumors called dimerizaing agent–regulated immunoreceptor complex (DARIC33). T cell products demonstrated target-specific and rapamycin-dependent cytokine release, transcriptional responses, cytotoxicity, and in vivo antileukemic activity in the presence of as little as 1 nM rapamycin. Rapamycin withdrawal paused DARIC33-stimulated T cell effector functions, which were restored following reexposure to rapamycin, demonstrating reversible effector function control. While rapamycin-regulated DARIC33 T cells were highly sensitive to target antigen, CD34^+^ stem cell colony-forming capacity was not impacted. We benchmarked DARIC33 potency relative to CD19 CAR T cells to estimate a T cell dose for clinical testing. In addition, we integrated in vitro and preclinical in vivo drug concentration thresholds for off-on state transitions, as well as murine and human rapamycin pharmacokinetics, to estimate a clinically applicable rapamycin dosing schedule. A phase I DARIC33 trial has been initiated (PLAT-08, NCT05105152), with initial evidence of rapamycin-regulated T cell activation and antitumor impact. Our findings provide evidence that the DARIC platform exhibits sensitive regulation and potency needed for clinical application to other important immunotherapy targets.

## Abstract 2

Low-nanomolar rapamycin concentration regulates the heterodimerization of CD33-specific DARIC T cells in preclinical models of acute myeloid leukemia.

## Graphical abstract

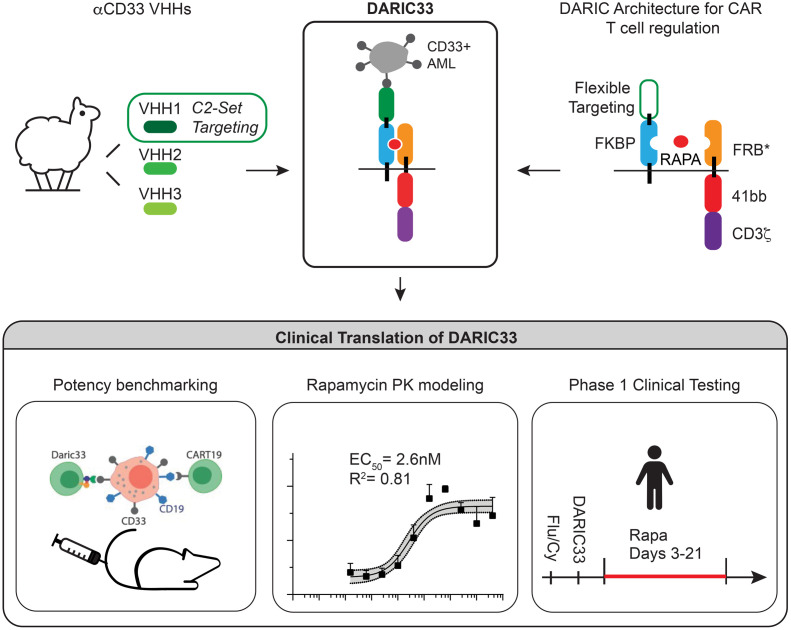

## Introduction

Chimeric antigen receptor (CAR) T cell products are potent living drugs that dramatically expand in the days following adoptive transfer into patients. For existing CD19 and BCMA CAR T cell products, T cell engraftment, expansion, function, and persistence characteristics are product-autonomous, such that, at their peak, CAR T cells may number 100- to 1,000-fold greater than the number of cells initially infused (1). Toxicities, such as cytokine release syndrome, immune effector cell–associated neurotoxicity syndrome, and rare late effects, such as marrow hypoplasia, have also been reported (2). Technologies that allow physicians to control the activity of engineered T cell therapies following patient infusion may address some of the safety concerns associated with this promising class of drugs.

In many applications, such as acute myeloid leukemia (AML) and solid tumors, on-target/off-tumor product reactivity may negatively impact therapeutic index. In the case of B cell malignancies, broad targeting and elimination of both normal and malignant CD19^+^ cells is generally well tolerated and clinically manageable. However, in the case of AML, no known target allows selective ablation of malignant myeloid cells without the simultaneous loss of essential nonmalignant cell types (3). Despite this caveat, hematopoietic stem/progenitor cell (HSPC) antigens with increased expression on AML blasts, such as CD33, CD123, and CLL1 (CLEC12A), have emerged as potential targets. Thus, targeting AML antigens with constitutively active CAR T cell products may eliminate HPSCs, resulting in prolonged or permanent marrow hypoplasia. A technological platform that allows for recursive cycles of tumor killing interspersed with periods of myeloid recovery, akin to cycles of cytotoxic chemotherapy, is a conceptually attractive approach to target this class of AML antigens.

Drug-induced dimerization of split CAR designs may be a general approach to allow physicians to modulate CAR activity in a time scale matched to clinical need. Previously reported dimerizing agent–regulated immunoreceptor complexes (DARICs) are composed of separate antigen targeting and T cell signaling components, with embedded extracellular rapamycin-dependent heterodimerizing domains (4). Targeting and signaling components dimerize in the presence of rapamycin, resulting in antigen-responsive T cell activation (4, 5). Prior studies demonstrated that CD19-targeted DARIC T cells (DARIC19) display off to on (e.g., quiescent to antigen-responsive) functional transitions in the presence of sub-immunosuppressive concentrations of rapamycin (e.g., ≤1 nM), well below the range recommended for immunosuppression in patients after solid organ transplant (6). Further, DARIC19 T cells in the presence of nanomolar concentrations of rapamycin exhibited potency equivalent to that of conventional CD19 CAR T cells in preclinical in vitro and in murine disease models.

Here, we describe the assembly and validation of a CD33-targeted DARIC chimeric immunoreceptor. From a single-domain V_H_H antibody library, we identified candidate CD33 binders that redirect T cell effector functions to target cells expressing CD33, an established AML antigen (7). Among several high-affinity single-domain antibodies, we identified one candidate capable of recognizing an epitope within the membrane-proximal C2-set domain of CD33, which is a conserved domain across CD33 splice isoforms (8). DARIC receptors incorporating the C2 epitope–specific V_H_H (DARIC-V_H_H1) displayed rapamycin-dependent recognition and activation against multiple gene-modified and AML cell lines, as well as an in vivo antitumor effect against established CD33^+^ human tumor xenografts in NSG mouse models. Following manufacture of clinical-scale DARIC33 T cell lots using good manufacturing practice (GMP) methodologies, we found that donor-matched DARIC33 T cells and control CD19 CAR T cells both exhibited similar expression of phenotypic markers associated with engraftment fitness while neither expressed markers associated with tonic signaling or exhaustion. Finally, correlation of dose-exposure and activity relationships establishes rapamycin concentration thresholds required for DARIC33 T cell activity in vivo. Based on these findings, we have initiated a phase I DARIC33 trial in pediatric patients with relapsed/refractory AML and demonstrated initial signs of rapamycin-mediated T cell activation and tumor response. Together, these observations will serve as a model for the development of additional drug-regulated T cell therapies.

## Results

**Figure 1:**
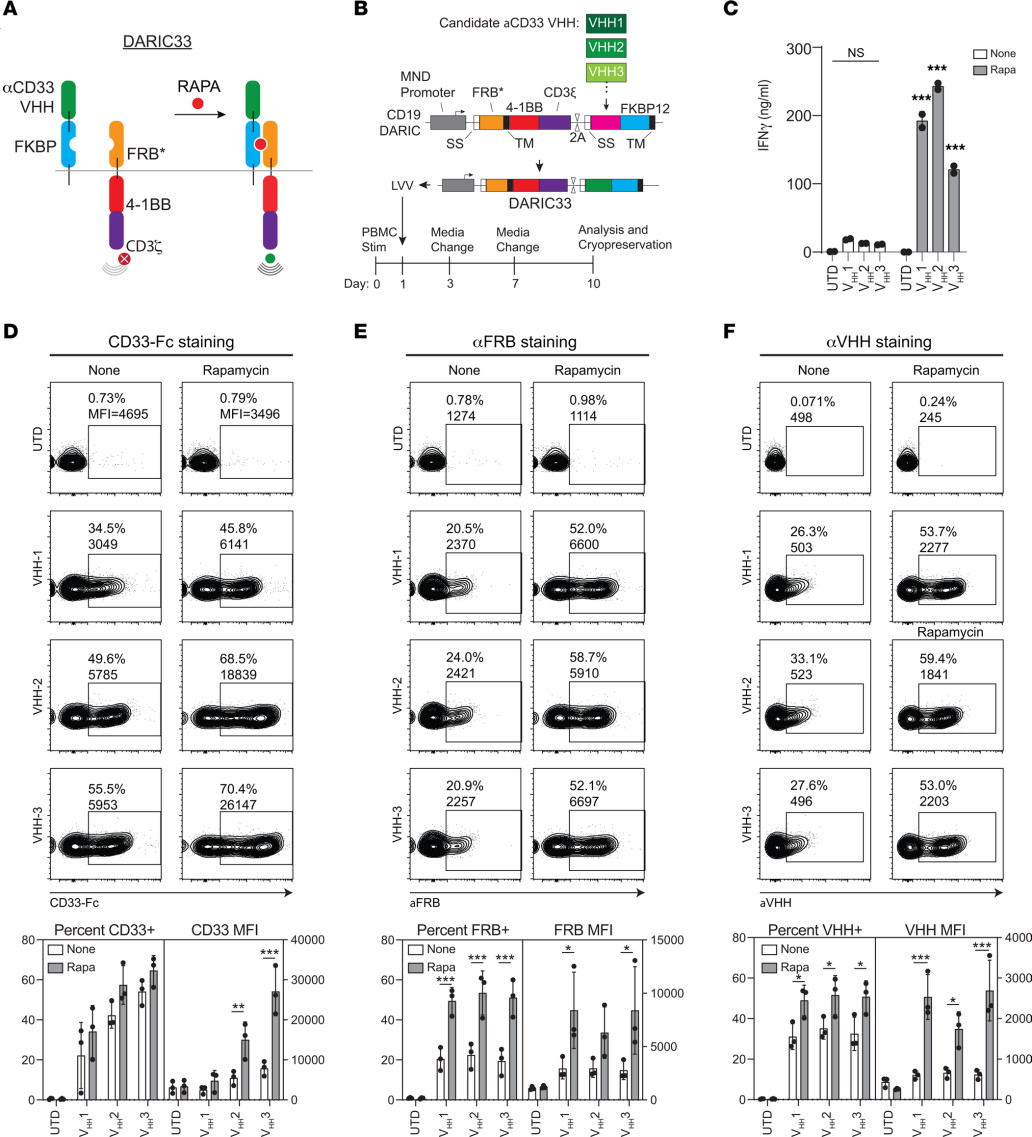
Rapamycin licenses permits antigen-dependent DARIC33 T cell responses and stabilizes surface expression of DARIC33 components. (**A**) Schematic depicting rapamycin-dependent activation of DARIC33. In the absence of rapamycin, the two DARIC components are split and do not respond to antigen. Following rapamycin addition, heterodimerization of DARIC components enables antigen-dependent T cell responses. (**B**) Schematic depicting generation of DARIC33 candidates and T cell production. DNA sequences encoding modified V_H_H sequences are incorporated into DARIC33 lentiviral expression vectors. (**C**) IFN-γ release by DARIC33 cell products following coculture with CD33^+^ MV4-11 AML cells. One of *n* = 3 donors is shown. ***P < 0.001, ANOVA with Tukey’s multiple-comparison correction. (**D**–**F**) Rapamycin stabilizes surface expression of DARIC33 components. DARIC33 cell products were cultured in medium alone or medium containing 1 nM rapamycin overnight before staining and evaluation by flow cytometry. Representative flow cytometry plots from 1 of 3 donors (above) with quantitation of percentage positive and median fluorescence intensity (MFI) from all 3 donors (below). *P < 0.05, **P < 0.01, ***P < 0.001, 2-way ANOVA with Šidák’s multiple-comparison correction, *n* = 3 donors. (**D**) Rapamycin increases antigen binding capacity of DARIC33 cells. (**E**) Rapamycin increases surface expression of the antigen signaling arm of DARIC. (**F**) Rapamycin increases surface expression of the antigen recognition arm of DARIC.

The DARIC architecture uses a split CAR design in which the antigen targeting and T cell signaling domains are separated into distinct transmembrane receptors that contain extracellular cognate rapamycin-dependent heterodimerization domains (Figure 1A). This bipartite design leverages a highly energetically favorable ternary complex between rapamycin and the rapamycin-binding domains derived from FK506-binding protein 12kd (FKBP12) and mammalian target of rapamycin (mTOR) FKBP12-rapamycin-binding (FRB) domain. The FRB domain was modified to incorporate the T2098L mutation, which destabilizes domain folding in the absence of rapamycin and increases the rate of protein turnover (9), promoting a stringent off state. We previously reported potent and reversible rapamycin-dependent antitumor responses by CD19-DARIC T cells (4) and sought to adapt this design for an AML therapeutic approach by engineering the DARIC to target CD33.

### Construction of rapamycin-dependent CD33-targeted DARICs.

To identify CD33-targeting single-domain antibodies, a library screen of heavy chain–only (V_H_H) binders isolated following alpaca immunization and yeast surface display was conducted. Three lead V_H_H candidates that bound recombinant human CD33 protein were identified. To confirm hits, binders were tested against CHO cells transiently expressing CD33 using increasing amounts of purified recombinant anti-CD33 V_H_H-Fc fusion proteins and secondary antibodies. Evaluation of binding isotherms revealed apparent affinities ranging from 0.9 nM to 249 nM (Supplemental Figure 1A; supplemental material available online with this article; https://doi.org/10.1172/JCI162593DS1). The binding characteristics of clone V_H_H1 were further characterized by surface plasmon resonance (Supplemental Figure 1B).

To determine whether CD33-specific V_H_H domains are capable of redirecting DARIC T cell effector functions, codon-optimized V_H_H domains were embedded as the targeting moiety of the DARIC architecture, resulting in DARIC-V_H_H1–3 (4). Peripheral blood mononuclear cells (PBMCs) were activated with CD3/CD28 antibodies, transduced with lentiviral vectors, and expanded for in vitro analysis (Figure 1B). T cell products contained an average of 1.5 to 2 integrated lentiviral genomes per cell, independent of construct (Supplemental Figure 1C). Untransduced (UTD) T cells, in both the presence and the absence of rapamycin, exhibited low or undetectable levels of interferon-γ (IFN-γ) release following coculture with CD33^+^ tumor cells in media with and without rapamycin (Figure 1C), reflecting minimal T cell activation. In addition, while none of the DARIC-V_H_H T cell products responded to CD33^+^ stimulator cells in the absence of rapamycin, each produced 120–240 μg/mL IFN-γ when 1 nM rapamycin was added to the coculture, an increase of 50- to 74-fold above UTD cells (Figure 1C). These data demonstrate that DARIC-V_H_H T cells are stringently dependent on rapamycin for effector cytokine release.

### Rapamycin stabilizes the surface expression of DARIC components.

The FRB domain has been shown to act as a rapamycin-sensitive degron (10). Therefore, we assessed the effect of rapamycin exposure on surface expression of both targeting and signaling polypeptide receptors on DARIC-V_H_H T cells. Following incubation of DARIC-V_H_H T cells in standard medium or medium containing 1 nM rapamycin, we evaluated surface expression of DARIC-V_H_H components using flow cytometry by staining cells with biotinylated CD33 antigen, anti-FRB, or anti-V_H_H antibodies. Across all DARIC-V_H_H constructs, rapamycin exposure increased the proportion of T cells binding soluble CD33 antigen by 26%–38% (ANOVA, *P* = 0.0232), and the median fluorescence intensity (MFI) 2- to 4-fold (ANOVA, *P* < 0.0001) (Figure 1D). We also observed increased surface expression of both FRB and V_H_H domains after rapamycin exposure (ANOVA, *P* < 0.001; Figure 1, E and F). While the percentage of DARIC^+^ cells diverged among the various detection methods, the MFI ratios with or without rapamycin were similar with all analytic approaches. In addition and similar to the results with DARIC19 (4), these results demonstrate that rapamycin increases surface expression of DARIC33 components to facilitate T cell responses.

### DARIC-V_H_H T cells are sensitive to low levels of rapamycin and CD33 antigen.

**Figure 2:**
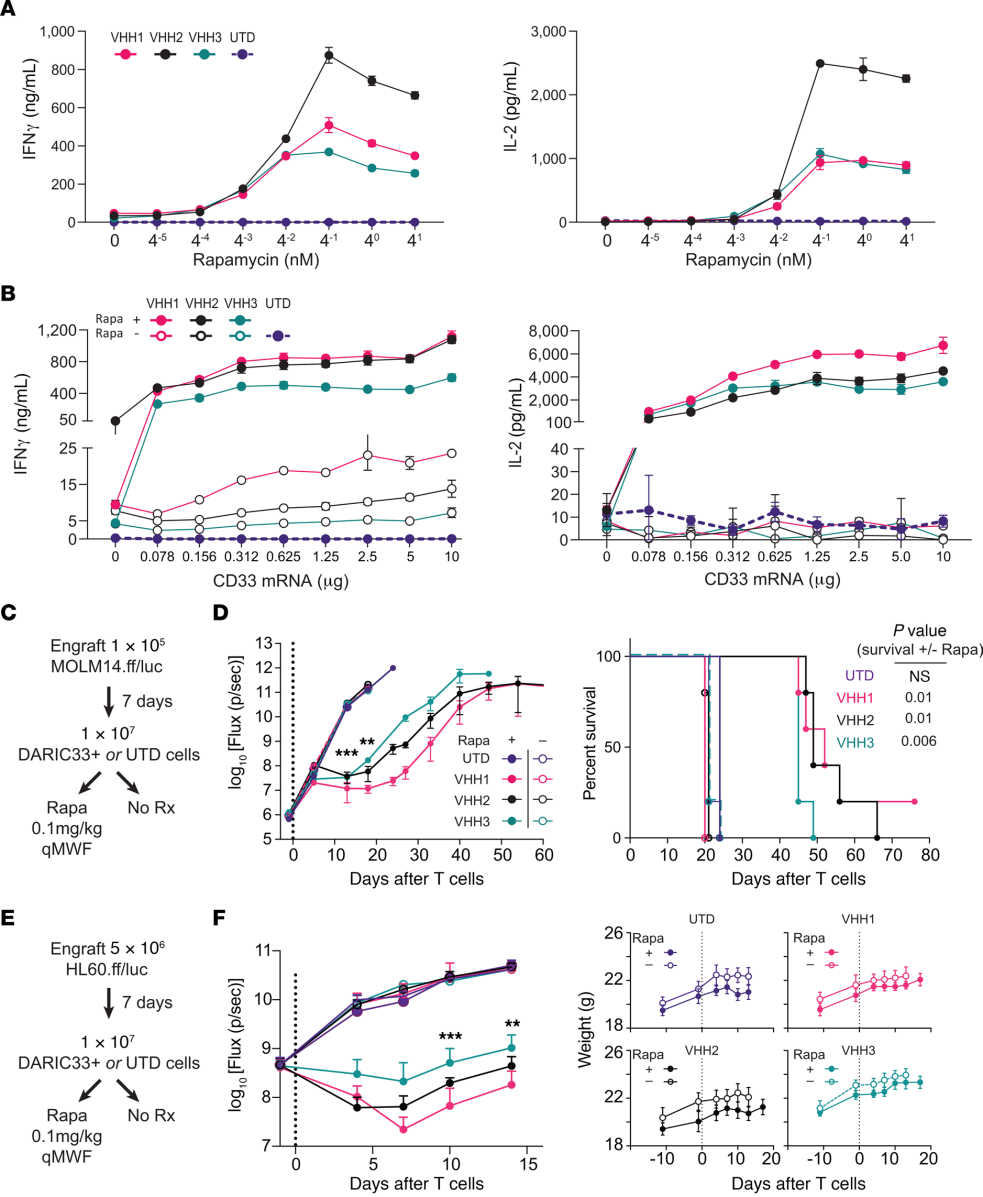
DARIC33-stimulated T cell responses require low levels of target antigen and low concentrations of rapamycin. (**A**) Cytokine release by DARIC33 cells following coculture with MV4-11 AML target cells in the presence of increasing concentrations of rapamycin. IFN-γ is shown at left, IL-2 at right. (**B**) Cytokine release by UTD (control) or DARIC33 cells following coculture with or without rapamycin and HEK293 T cells electroporated with increasing amounts of CD33 mRNA. (**C** and **D**) 10^7^ DARIC33^+^ cells or an equivalent number of UTD control cells were infused intravenously in NSG mice 7 days after engraftment of 1 × 10^5^ MOLM14.ff/luc leukemia cells per animal. After T cell infusion, mice were treated 3 times per week with 0.1 mg/kg rapamycin or were observed. (**D**) Left: Quantification of tumor growth by BLI; mean ± SEM, *n* = 5 mice per group. Right: Symptom-free survival with comparisons by Mantel-Cox (log-rank) test. (**E** and **F**) 10^7^ DARIC33^+^ cells or an equivalent number of UTD control cells were infused intravenously in NSG mice 7 days after engraftment of 5 × 10^6^ HL-60.ff/luc leukemia cells per animal. After T cell infusion, mice were treated 3 times per week with 0.1 mg/kg rapamycin or were observed. (**F**) Left: Quantification of tumor growth by BLI; mean ± SEM, *n* = 5 mice per group. Right: Mouse weight. Time points at which all DARIC33 formats meet the *P* value threshold when compared with UTD cells plus rapamycin (**D** and **F**) are indicated as **P < 0.01, ***P < 0.001, using repeated-measures ANOVA with Dunnett’s multiple-comparison correction.

To determine rapamycin concentration thresholds required for DARIC-V_H_H T cell activation, we assayed cytokine release from 24-hour cocultures of DARIC-V_H_H T cells and CD33^+^ AML target cells, including a rapamycin concentration range up to 4 nM (Figure 2A). Release of IFN-γ and IL-2 followed a sigmoidal response to increasing rapamycin concentrations, reaching a maximum in the presence of 0.25 nM rapamycin and remaining unchanged at higher rapamycin concentrations. DARIC-V_H_H2 induced more cytokine release than the other two V_H_H clones. The rapamycin EC_50_ for DARIC-V_H_H T cell activation, determined by cytokine release, ranged from 15.8 pM to 74.2 pM for IFN-γ and IL-2 (Figure 2A), and 17 pM to 52 pM for TNF-α (Supplemental Figure 2A). Based on these data, the rapamycin concentration required for DARIC-V_H_H T cell activation in the presence of CD33^+^ target cells is near or below the IC_50_ of mTORC1 (62 pM) or mTORC2 (534 pM) (11).

We next assessed CD33 antigen sensitivity by coculturing DARIC-V_H_H T cells with target cells expressing a range of CD33 antigen densities in the presence or absence of rapamycin. HEK293T cells electroporated with escalating amounts of CD33 mRNA exhibited dose-dependent levels of cell surface CD33 protein as determined by flow cytometry (Supplemental Figure 2B). In the presence of rapamycin and target cells with increasing CD33 antigen density, IFN-γ release by DARIC-V_H_H T cells increased between 19- and 140-fold and IL-2 release increased between 408- and 618-fold (Figure 2B). Among samples treated with CD33 mRNA, CD33 expression increased 38-fold, while IFN-γ release by DARIC-V_H_H T cells increased only 2- to 3-fold, suggesting saturation of DARIC-V_H_H signaling outputs at low densities of CD33. We further evaluated rapamycin-dependent induction of IFN-γ release following stimulation of DARIC-V_H_H T cells by target cells expressing lower densities of CD33 (Supplemental Figure 2, D and E). One construct, DARIC-V_H_H2, released substantial amounts of IFN-γ in the presence of rapamycin and unmanipulated HEK293T cells, suggesting antigen-independent signaling of this construct (Figure 2B and Supplemental Figure 2E). Addition of soluble CD33 protein to coculture experiments did not inhibit rapamycin-dependent DARIC33 stimulation of T cell responses (Supplemental Figure 2F). Together these results demonstrate that rapamycin-activated DARIC-V_H_H T cells exhibit sensitivity to AML target cells with low CD33 densities.

### DARIC-V_H_H T cells exhibit rapamycin-dependent antileukemic activity in vivo.

To evaluate antitumor activity in vivo, we used xenograft tumor models in which immunodeficient NSG mice are intravenously inoculated with luciferase-tagged AML cell lines in the context of a range of rapamycin doses and administration schedules. MOLM14, a cell line derived from a secondary AML, exhibits robust CD33 expression (Supplemental Table 1) and, following modification for bioluminescence imaging (BLI), grows rapidly when inoculated into NSG mice. After intravenous inoculation of MOLM14 AML cells, we treated mice with DARIC-V_H_H or UTD control T cells followed by rapamycin delivered at a dose of 0.1 mg/kg by intraperitoneal injection 3 times weekly for the duration of the study (Figure 2C). Mice treated with UTD T cells demonstrated logarithmic increases in tumor burden and developed tumor-associated symptoms within 3 weeks (Figure 2D). Mice treated with DARIC-V_H_H T cells without rapamycin exhibited equally rapid tumor progression. In contrast, mice treated with DARIC-V_H_H T cells and rapamycin displayed delayed tumor growth and significantly extended survival.

As a second xenograft tumor model to evaluate DARIC-V_H_H antitumor activity, we used the CD33-expressing acute promyelocytic leukemia-like cell line HL-60, modified for BLI (Figure 2E). We similarly observed DARIC-V_H_H T cell antitumor activity that was fully rapamycin dependent (Figure 2F). None of the mice in either HL-60 or MOLM14 models lost weight following adoptive transfer of DARIC-V_H_H T cells, either alone or followed by rapamycin administration (Figure 2F and data not shown). Across both AML tumor models, the rank order of antitumor activity exhibited by the DARIC-V_H_H constructs was preserved. Together, these studies demonstrate that the DARIC-V_H_H chimeric immunoreceptor architecture elicits in vivo antitumor activity in the presence of rapamycin.

### DARIC-V_H_H1 T cell activation is specific to the membrane-proximal domain of CD33.

Because alternative splicing impacts CD33 expression (8, 12, 13), we included cDNAs encoding both full-length CD33M and the major alternative shorter transcript CD33m, which lacks the membrane-distal Ig-like IgV2 sialic acid–binding domain encoded by exon 2 (14–16), in our screening library. Expression of CD33M resulted in a strong fluorescent signal following staining with all three V_H_H-Fc fusion proteins, while expression of CD33m resulted in a strong fluorescent signal only following staining with V_H_H1-Fc (Supplemental Figure 1A and Supplemental Figure 3A). To verify the CD33 membrane-proximal epitope specificity, we stained CHO cells transiently expressing CD33m with increasing concentrations of purified V_H_H-Fc fusions and secondary antibodies. V_H_H1-Fc fusion bound CD33m-expressing CHO cells with an apparent *K_D_* = 162 nM, whereas no binding of V_H_H2-Fc or V_H_H3-Fc to CD33m was detected (Supplemental Figure 3A).

**Figure 3:**
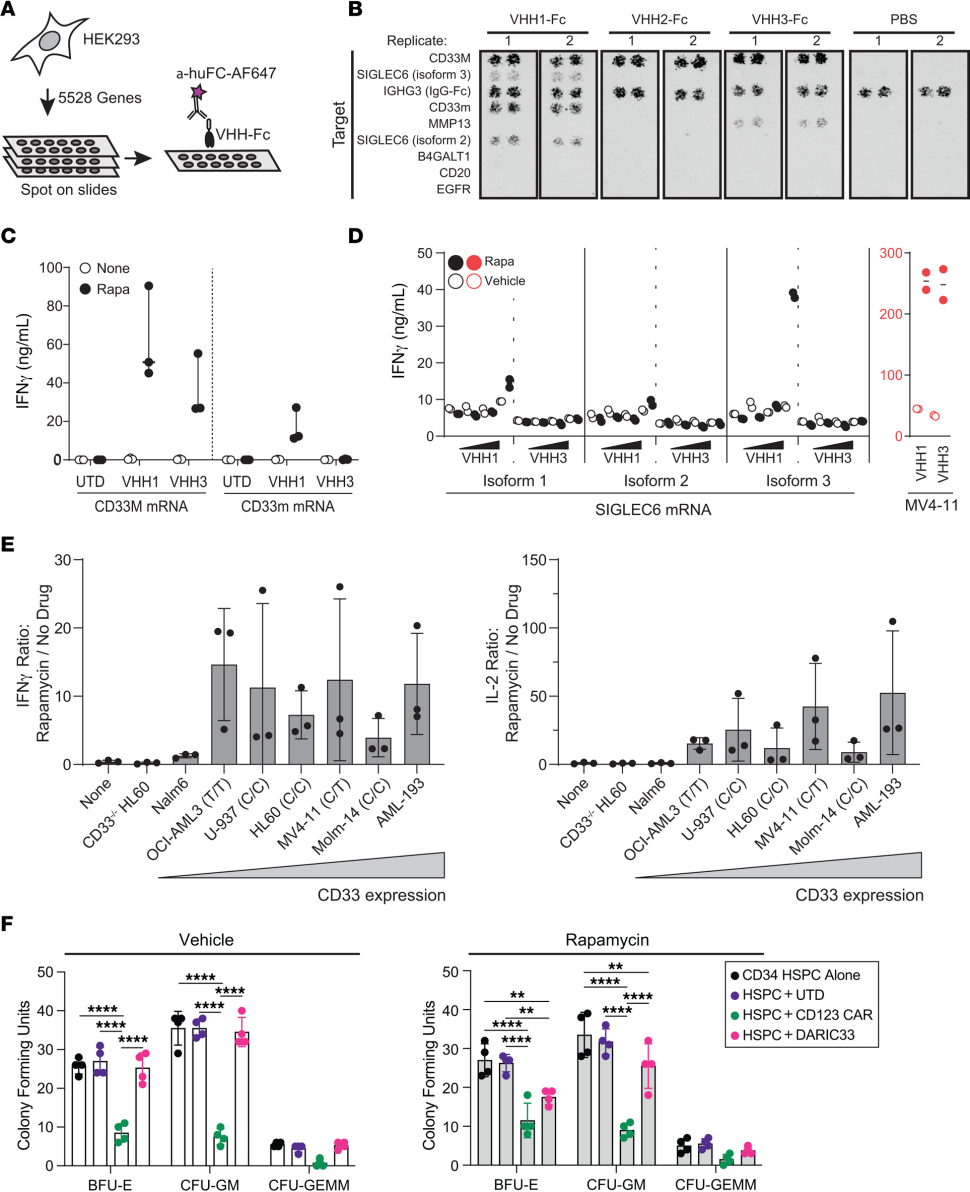
DARIC33 is specific for CD33 antigen and does not inhibit HSPC colony formation. (**A** and **B**) Evaluation of CD33-specific V_H_H-Fc fusion proteins used in DARIC33 designs. (**A**) Schematic depicting detection strategy of V_H_H-Fc fusions binding to HEK293 cells expressing one of 5,528 surface-bound or secreted proteins. After reverse transfection, HEK293 cells are spotted onto slides, then stained with V_H_H-Fc proteins (or PBS control) and Alexa Fluor 647–labeled anti–human Fc secondary antibodies. (**B**) Secondary screen of selected hit and control transgenic HEK293 samples (*n* = 2 replicates shown). (**C**) Stimulation of T cell IFN-γ release by DARIC33 designs in the presence of rapamycin following exposure to HEK293 cells electroporated with mRNA encoding CD33M (left) and CD33m (right). (**D**) Left: Stimulation of T cell IFN-γ release by DARIC33 designs in the presence of rapamycin following exposure to HEK293 cells electroporated with mRNA encoding Siglec-6. Right: Release of IFN-γ following coculture of DARIC33 with MV4-11 AML cells is shown for comparison. (**E**) Correlation of CD33 density (expressed as the logarithm of the antigen binding capacity) with release of IFN-γ (left) and IL-2 (right). (**F**) Colony-forming units following culture of CD34^+^ cells alone or with T cells in the presence or absence of rapamycin. Colonies were enumerated after 14 days of growth. *n* = 2 T cell donors. **P < 0.01, ****P < 0.0001, ANOVA with Tukey’s multiple-comparison correction.

To evaluate potential off-target activity, we screened CD33-targeting V_H_H domains for binding to a library of transgenes encoding 5,528 secreted and transmembrane proteins. HEK293T cells expressing library transgenes were spotted on slides, fixed, and stained with anti-CD33 V_H_H domain–Fc protein fusions followed by fluorescently labeled anti-Fc secondary antibodies (Figure 3A). This screen did not identify strong binding of the CD33-specific V_H_H clones to non-CD33 cell surface molecules in the library. We did observe weak fluorescent signal of V_H_H1 toward samples expressing Siglec-6 (RefSeq accession NM_198845), a sialic acid–binding protein recently identified as a potential antigen target for AML (17) that shares substantial homology with CD33. We also observed weak V_H_H3 reactivity toward MMP-13 (Figure 3B).

To determine whether DARIC-V_H_H1 T cells respond to CD33m or Siglec-6, we cultured DARIC T cells with HEK293T cell lines electroporated with CD33M-, CD33m-, or Siglec-6–encoding mRNA (Figure 3, C and D). Consistent with the findings above, DARIC-V_H_H1 T cells exhibited rapamycin-dependent IFN-γ release following coculture with HEK293T cells expressing CD33M or CD33m, whereas T cells expressing DARIC-V_H_H3 T cells responded only to HEK293T cells expressing CD33M (Figure 3C). Similarly, we cultured DARIC-V_H_H1 or DARIC-V_H_H3 T cells with HEK293T cells, electroporated with titrated amounts of Siglec-6 mRNA, in the presence or absence of rapamycin (Figure 3D). When rapamycin was present, HEK293T cells electroporated with the highest amounts of Siglec6 mRNA stimulated release of 40–60 ng/mL IFN-γ from DARIC-V_H_H1 T cells, which corresponds to approximately 10% of the amount released following coculture with CD33^+^ AML cells. No IFN-γ release was observed when DARIC-V_H_H1 T cells were cultured with HEK293T cells expressing lower levels of Siglec-6. Transgenic expression of CD33 in lung cancer cells that do not endogenously express CD33 (Supplemental Figure 3B) resulted in rapamycin-dependent DARIC-V_H_H1 T cell proliferation in vitro, whereas targeted deletion of CD33 from CD33^+^ AML cell lines eliminated IFN-γ responses in vitro (Supplemental Figure 3, C and D) and in vivo (Supplemental Figure 3G). In addition, DARIC-V_H_H1 T cells exhibited antitumor activity in CD33^lo^ Nalm6 xenograft tumor models (Supplemental Figure 3, E and F). Together, these data show that stimulation of T cell effector functions by DARIC-V_H_H1 is specific to the membrane-proximal domain of CD33 present in both CD33M and CD33m isoforms. The strict target specificity, promising affinity characteristics, rapamycin dependence, and recognition of both CD33M and CD33m led us to select DARIC-V_H_H clone 1 as a lead clinical candidate for subsequent development. Below, we refer to DARIC-V_H_H1 simply as DARIC33, and T cell products manufactured by Seattle Children’s Therapeutics at clinical scale using GMP-compatible reagents and techniques as SC-DARIC33.

### CD33m is a prevalent isoform of CD33 expressed by AML.

Four separate single-nucleotide polymorphisms (SNPs) have been reported to influence splicing of CD33 by SRSF2 (14, 16), including rs12459419 C>T in the splice enhancer region that regulates exon 2 skipping and rs2455069 A>G resulting in protein modification (14–16). Though controversial (18), the rs12459419 T/T genotype has been associated with predominance of the shortened transcript (lacking exon 2) encoding CD33m, and poor responses to CD33-targeted therapeutics that recognize the IgV2 domain missing from CD33m (14, 19). We reviewed transcriptional profiles of 577 AML cases, evaluating the proportion of CD33 transcripts lacking exon 2. We identified strong correlations between SNPs and CD33m transcript expression among AML cases (Supplemental Figure 4, A–E). We also identified CD33m transcripts among profiles of healthy tissues, though with less abundance than among AML cases (Supplemental Figure 4F). While attempts at targeting the CD33m isoform for AML immunotherapy are being developed (20, 21), these strategies have also faced challenges. As a potential control, some studies have shown that antibody HIM3-4 is specifically reactive to the CD33m isoform; we observed minimal reactivity of this clone toward CD33m-overexpressing cells (Supplemental Figure 4G). Coculture of DARIC33 T cells with AML cells of various rs12459419 SNP genotypes (21, 22) including OCI-AML3 (T/T), U-937 (C/C), HL-60 (C/C), MV4-11 (C/T), and MOLM14 (C/C) stimulated similar rapamycin-dependent release of IFN-γ and IL-2 (Figure 3E), despite different CD33M expression density (assessed using IgV2-targeted p67.6 antibody; Supplemental Table 1). Combined, these findings demonstrate the challenges associated with targeting the CD33m epitope with established antibody clones and support the use of DARIC33 to target CD33^+^ cells across a range of expression and isoform usage.

### DARIC33 T cells do not impact hematopoietic colony-forming capacity.

CD33 is expressed by granulocyte precursors (23) as well as hematopoietic stem/progenitor cells (HSPCs) with potential for multilineage engraftment in immunodeficient mice (24). Elimination of HSPCs may result in intolerable myeloablation (25, 26). However, recent studies found that the number of cells with the potential to form multilineage colonies in stem cell plating assays is not decreased by exposure to CD33 CAR T cells (27). To assess hematopoietic safety of DARIC33, we plated purified CD34^+^ HSPCs in colony-forming assays following overnight incubation alone or together with a 10-fold excess of DARIC33 cells or comparator T cell products and rapamycin. As expected, colony-forming units (CFU) of the granulocyte/monocyte lineage (CFU-GM) and of multilineage precursors (granulocyte-erythrocyte-monocyte-megakaryocyte CFU [CFU-GEMM]) were markedly reduced following incubation with CD123 CAR T cells (28), but not following incubation with UTD control cells (Figure 3F). Compared with UTD T cells, the numbers of CFU-GM and erythroid burst-forming units (BFU-E) were slightly reduced following coculture with DARIC33 effector T cells in the presence of rapamycin, but not when rapamycin was omitted from the overnight culture. These data suggest that while activated DARIC33 T cells have some impact on hematopoietic colony formation, this is a rapamycin-dependent process that can be controlled by withdrawal of the drug.

### Rapamycin exposure drives an antigen-dependent CAR T cell activation signature in DARIC33 T cells.

**Figure 4:**
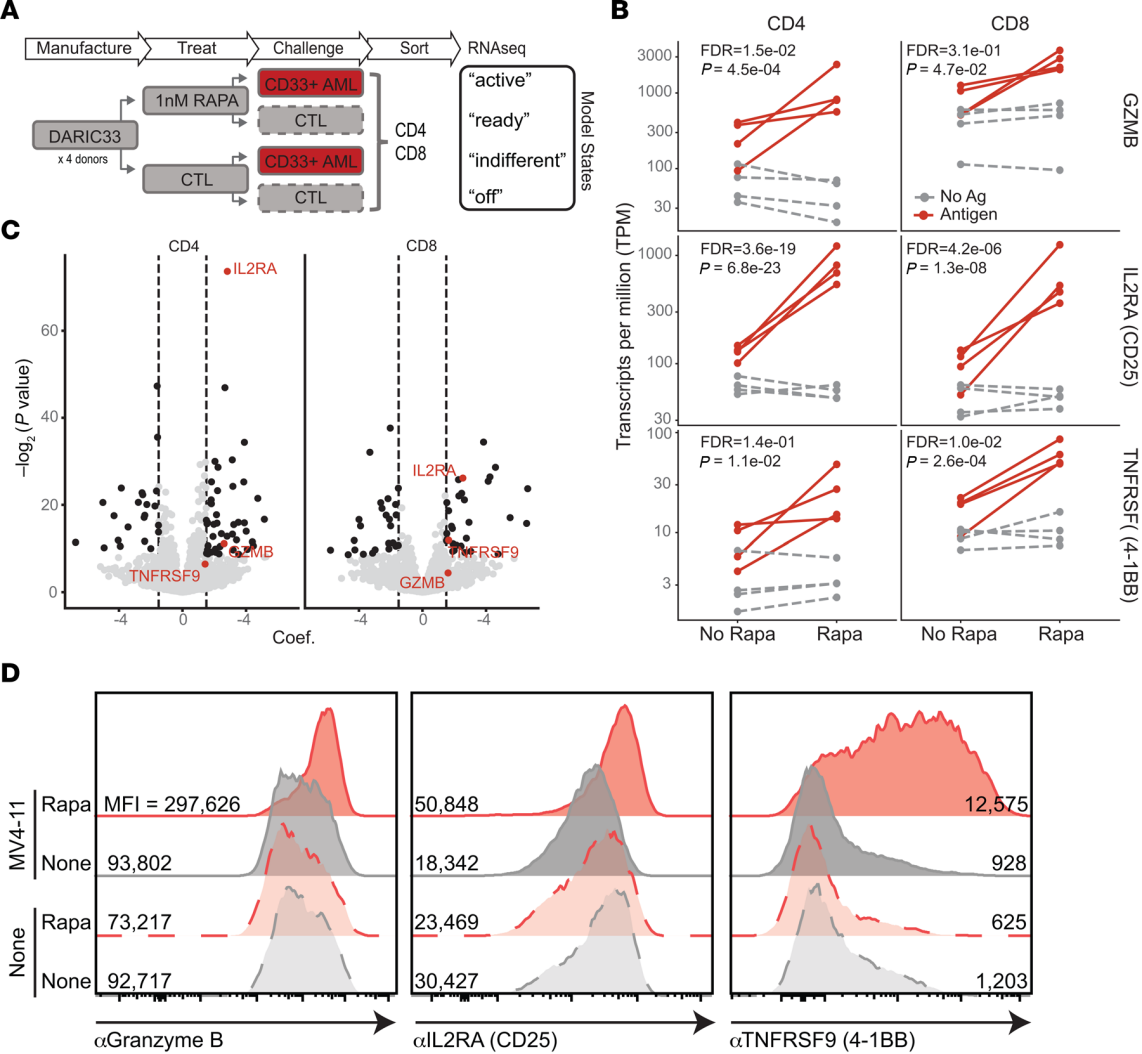
DARIC33 stimulates T cell transcriptional responses in the presence of antigen and rapamycin and without hallmarks of tonic signaling. (**A**–**D**) DARIC33 cells derived from *n* = 4 healthy donors were incubated with 1 nM rapamycin or medium alone before culture alone or with CD33^+^ MV4-11 AML target cells. After coculture, CD4^+^ and CD8^+^ cells were sorted and evaluated by RNA-Seq. (**A**) Schema for the experiment. DARIC33 cells resting in the absence of rapamycin or antigen are considered “off,” whereas DARIC33 cells incubated in rapamycin without antigen and with antigen exposure are labeled “ready” and “active,” respectively. (**B**) Transcriptional responses among selected genes associated with early T cell activation. (**C**) Volcano plot of the magnitude of statistical significance (*y* axis) versus magnitude of rapamycin and antigen (e.g., “DARIC-active”) effect (*x* axis, labeled “Coef.” in the figure). GZMB, IL2RA, and TNFRSF9 are shown in red, and additional genes exhibiting significant “DARIC-active” regulation are shown in black, with more detail provided in a heatmap shown in Supplemental Figure 5. (**D**) Flow cytometric confirmation that transcriptional changes are reflected in protein abundance. MFI for each sample is shown.

Transcriptional programs are tightly associated with T cell differentiation and functional status (29, 30). We interrogated transcriptional changes of sorted CD4^+^ and CD8^+^ DARIC33 cells following antigen exposure in the presence or absence of rapamycin (see Figure 4A for schema). We then modeled transcriptional changes to identify a “DARIC-active” profile distinct from either “antigen without rapamycin” or “rapamycin-only” transcriptional profiles, restricting our analysis to a subset of genes informative of changes in T cell states (see Supplemental Methods). Of the 2,792 queried genes, 228 genes showed transcriptional regulation specific to the DARIC-active condition in either the CD4^+^ or the CD8^+^ population, or both (genes with FDR <0.05 and fold change >2.8 over the combined individual effects of rapamycin and antigen; Figure 4B; see also Supplemental Figure 5). Following rapamycin exposure and antigen stimulation, DARIC33 cell transcriptional profiles showed significant enrichment of CAR T cell activation genes (Fisher’s exact test, CD4 *P* = 0.024 and CD8 *P* = 0.005), including *GZMB*, *IL2RA*, and *TNFRSF9* (encoding 4-1BB) (Figure 4C), which was also reflected by changes in protein abundance as measured by flow cytometry (Figure 4D). Taken together, these results reveal a transcriptional activation signature of DARIC33 CD4^+^ and CD8^+^ T cells in the presence of both target antigen and rapamycin. Notably, this signature is consistent with conventional CAR T cells activated by antigen in the absence of rapamycin (31–33).

### DARIC33 and CAR33 T cells have similar functionality and activation signature.

We have previously shown equivalent functional activity when the same CD19-targeting scFv was placed in a CAR versus a DARIC backbone (4). To investigate whether the CD33-targeting V_H_H1 also exhibited similar functional activity when placed in a CAR or DARIC backbone, we generated CD33-targeting CAR T cells using the identical CD33-specific V_H_H1 binder. Both CAR33 and DARIC33 T cells had similar expression and virus integration profile (Supplemental Figure 6A). When cocultured with CD33^+^ HL-60 tumor cells, CAR33 T cells had robust IFN-γ production in the presence or absence of rapamycin, while DARIC33 only secreted cytokines in the presence of rapamycin (Supplemental Figure 6B). In addition, both CAR33 and rapamycin-exposed DARIC33 T cells had similar rates of cytotoxicity in vitro (Supplemental Figure 6C). Next, we analyzed the phenotype of both CAR33 and DARIC33 T cells with or without activation. We observed some evidence of tonic signaling in the CAR33 T cells, characterized by increased CD69 and CD25 expression in comparison with unstimulated DARIC33 T cells (Supplemental Figure 6, D and E). Following coculture with CD33^+^ tumor cells, both CAR33 and DARIC33 T cells exhibited a similar activation profile; however, the CAR33 cells had higher expression of PD-1, LAG3, CD69, and CD25, suggesting greater activation following T cell activation (Supplemental Figure 6, D and E). Together, these data suggest similar rates of tumor reactivity for both CAR33 and DARIC33 platforms, with the DARIC33 cells demonstrating lower rates of tonic signaling compared with the CAR33 platform.

### GMP manufacturing at scale generates SC-DARIC33 cell products with features similar to those of CD19 CAR T cell products.

**Figure 5:**
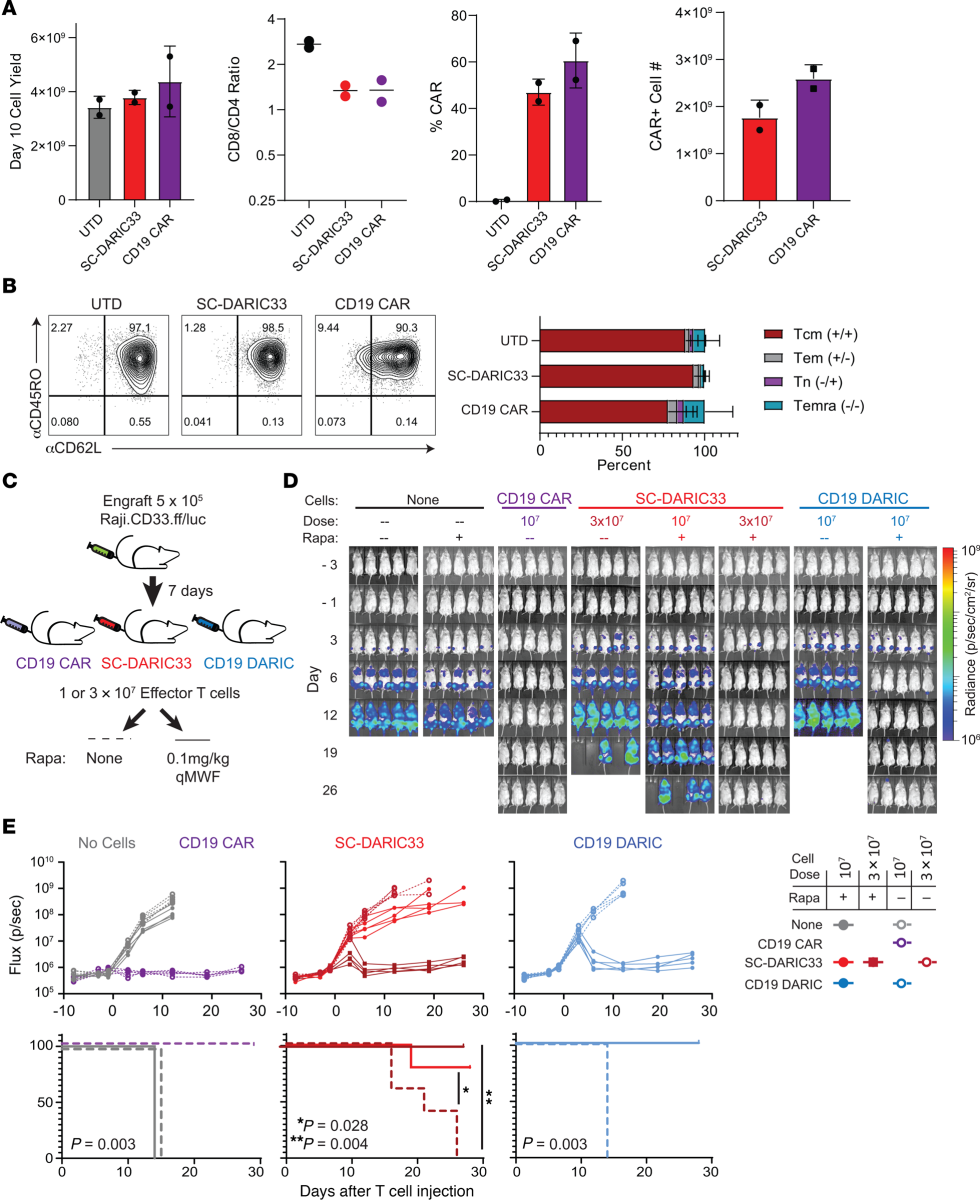
Clinically appropriate manufacture of donor-matched DARIC33 and CD19 CAR T cells allows comparisons of manufacture feasibility and cell potency. (**A**) Yields of UTD, CD19 CAR, and DARIC33 cell products following manufacture using reagents and techniques appropriate for clinical application from *n* = 2 donors. (**B**) Surface expression of CD45RO and CD62L of clinical cell product facsimiles. Representative flow plot is shown at left, with quantitation from *n* = 2 donors shown at right (stacked bars indicate mean ± SD). (**C**–**E**) 1 × 10^7^ to 3 × 10^7^ DARIC33^+^ cells, CD19 CAR T cells, or CD19 DARIC^+^ cells or an equivalent number of UTD control cells were infused intravenously in NSG mice 7 days after engraftment of 5 × 10^5^ Raji.CD33.ff/luc leukemia cells. After T cell infusion, mice were treated with 0.1 mg/kg rapamycin 3 times weekly for the indicated durations or were observed. (**C**) Schematic depicting experimental design. To compare cell potency with benchmark immunotherapy products, 2 doses of DARIC33^+^ cells were used. (**D**) Tumor progression monitored by bioluminescence; *n* = 5–8 mice per group. (**E**) Top: Quantitation of tumor growth, with points representing measurements of individual mice. Bottom: Kaplan-Meier survival estimates; log-rank test *P* values.

To evaluate the performance of DARIC33 generated at clinical scale (SC-DARIC33), we generated donor-matched (*n* = 2) SC-DARIC33 and CD19 CAR T cells using designs and manufacturing methods previously deployed in clinical trials at Seattle Children’s Hospital. In these trials, CD19 CAR T cell administration resulted in complete remission rates of more than 90% in children and young adults with relapsed or refractory B cell malignancies (34, 35). Compared with control CD19 CAR T cell products, SC-DARIC33 showed similar expansion kinetics, CD4/CD8 ratios, proportions of CAR/DARIC^+^ cells, and CAR/DARIC cell yields (Figure 5A). The frequency of cells expressing both CD62L^+^ and CD45RO^+^, a phenotype associated with preserved engraftment fitness and antitumor potential (36), was greater than 90% within each of the UTD, CD19 CAR, and SC-DARIC33 T cell products (Figure 5B).

Previous preclinical studies performed by our group found control of Raji xenograft tumor burden progression correlated with clinical activity of CD19 CAR T cell designs (37). To compare antitumor activity across CD19- and CD33-targeted T cell therapies, we generated Raji cells with matched levels of CD19 and CD33 expression via lentiviral transduction (Raji.CD33.ff/luc). Progression of intravenously injected Raji.CD33.ff/luc xenograft tumor burden was monitored following administration of CD19 CAR, SC-DARIC33 (tested at 2 doses), and CD19-specific DARIC T cell products manufactured using a GMP process (see Figure 5C for schema). As expected, no Raji tumor burden progression was observed following infusion of 1 × 10^7^ CD19 CAR T cells per animal, whereas mice receiving no treatment or rapamycin alone (0.1 mg/kg qMWF delivered by intraperitoneal injection) showed rapid tumor growth and developed tumor-associated symptoms requiring euthanasia within 12 days (Figure 5D). Infusion of either 3 × 10^7^ or 1 × 10^7^ SC-DARIC33 T cells followed by rapamycin dosing suppressed tumor growth and prolonged survival of mice compared with control animals not receiving rapamycin (*P* = 0.028 and *P* = 0.004; Figure 5E). Administration of CD19-specific DARIC cells also resulted in rapamycin-dependent tumor suppression and prolonged survival in the Raji xenograft tumor model. Together, these data suggest that SC-DARIC33 may require higher T cell doses to achieve potency similar to that of CD19 CAR T cells.

### DARIC33 and SC-DARIC33 T cells display recursive rapamycin-dependent on-off-on functional state transitions.

**Figure 6:**
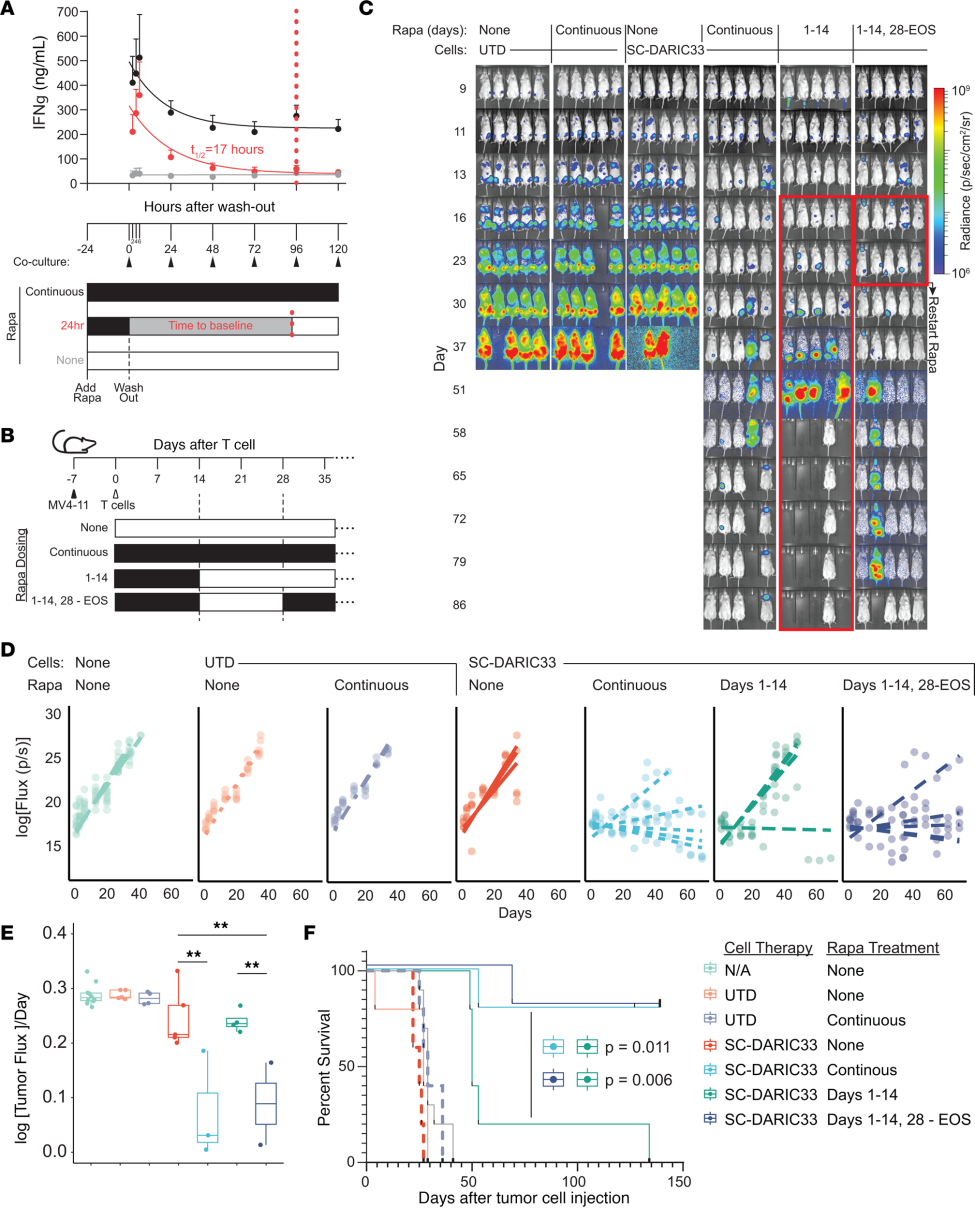
Activation of SC-DARIC33 is reversible. (**A**) DARIC33 cell cytokine responses to antigen at various times following washout from rapamycin-containing medium. DARIC33 cells replaced into rapamycin-containing medium or DARIC33 cells previously cultured in medium not containing rapamycin were used as comparators. The *t*_1/2_ was determined by fitting of a single-phase exponential decay. (**B**–**F**) 10^7^ SC-DARIC33^+^ cells or an equivalent number of UTD control cells were infused intravenously in NSG mice 7 days after engraftment of 1 × 10^6^ MV4-11.ff/luc leukemia cells. After T cell infusion, mice were treated with 0.1 mg/kg rapamycin 3 times weekly for the indicated durations or were observed. (**C**) Tumor progression monitored by bioluminescence; *n* = 5 mice per group. Images taken during a “pause” in rapamycin dosing are outlined in red. (**D**) Quantitation of tumor growth. Points are measurements of individual mice, lines are best-fit tumor growth trajectories (see Supplemental Methods). (**E**) Tumor growth rates. Points are growth rates fit for individual mice; boxes and whiskers show mean and SD. **P < 0.01, 2-tailed *t* tests with Benjamini-Hochberg correction for multiple comparisons. (**F**) Survival after infusion of DARIC33 cells or UTD cells followed by treatment with various rapamycin schedules. Mantel-Cox log-rank *P* values are shown uncorrected.

The capacity to temporarily pause DARIC T cell effector function in patients following SC-DARIC33 administration represents a potential control feature for mitigating potential toxicities and permitting hematopoietic recovery. Moreover, therapeutic T cells that are intermittently rested may be less prone to functional exhaustion and capable of repopulating memory cell compartments (29). We therefore developed systems to probe pharmacologic control of DARIC33 T cells. Kinetic assessments of DARIC33-induced cancer cell cytotoxicity showed rapid and complete rapamycin-dependent cancer cell killing only when the target antigen CD33 was expressed in cancer cells (Supplemental Figure 7, A and B). Killing rates increased immediately following rapamycin addition, and were maximal after 37 hours (Supplemental Figure 7, C and D). To define kinetic effects of rapamycin removal, DARIC33 T cells cultured with rapamycin for 24 hours were washed and rested for increasing periods of time in rapamycin-free medium before challenge with CD33^+^ MV4-11 AML target cells. At early time points, preactivated SC-DARIC33 T cells showed high levels of IFN-γ release. Increasing durations of rest resulted in a progressive decline of IFN-γ release that returned to baseline after 96 hours, following first-order kinetics characterized by a half-life of 17 hours (Figure 6A).

To evaluate the reversibility of SC-DARIC33 T cell activation in vivo, we treated mice bearing AML xenografts derived from MV4-11 cells modified for BLI with SC-DARIC33 T cells and rapamycin delivered following continuous (days 1–150), interrupted (days 1–14 and 28–150), or abbreviated (days 1–14) schedules (see Figure 6B for schema). Mice receiving UTD control cells (with or without rapamycin) exhibited tumor growth and tumor-associated symptoms by day 50, whereas mice treated with SC-DARIC33 T cells and rapamycin exhibited delayed tumor burden progression (Figure 6C), and prolonged symptom-free survival (Figure 6F). Four of five mice receiving the abbreviated rapamycin schedule exhibited tumor relapses approximately 21 days after rapamycin was discontinued. In contrast, when rapamycin was reinitiated on day 28, 4 of 5 mice controlled the tumor through the end of the observation period. Estimates of tumor growth kinetics, modeled using linear mixed effects, revealed similar tumor growth rates in negative control groups and animals treated with SC-DARIC33 and abbreviated rapamycin (Figure 6, D and E). However, among animals receiving either continuous or intermittent rapamycin, SC-DARIC33 T cells suppressed tumor growth rates and extended survival (Figure 6F). Together, these data are consistent with a model wherein discontinuation of rapamycin pauses SC-DARIC33 antitumor activity, which may be restored by resumption of rapamycin administration.

### Preclinical models define blood rapamycin concentrations associated with SC-DARIC33 activation in vitro and in vivo.

To support rapamycin dose selection for first-in-human testing of SC-DARIC33, we sought to define rapamycin concentrations required for DARIC33 activation both in vitro and in a mouse xenograft tumor model. We used Förster resonance energy transfer (FRET) to characterize the dimerization kinetics of the DARIC33 system in vitro. A PE-labeled anti-V_H_H antibody was used as a donor fluorophore, while an Alexa Fluor 647–labeled anti-FRB antibody was used as an acceptor (Supplemental Figure 8B). Labeling of DARIC33 cells with both antibodies, but not with either antibody alone, resulted in rapamycin-dependent fluorescence emission signal in the PE-Cy5 channel (Supplemental Figure 8A), indicating heterodimer-dependent FRET. We quantified rapamycin-mediated dimerization parameters by culturing DARIC33 cells in a gradient of rapamycin concentrations in medium and determined that the rapamycin EC_50_ for DARIC33 dimerization was 135 pM in T cell medium (Supplemental Figure 8C). To determine the time between rapamycin dosing and peak DARIC33 activation, we cultured DARIC33 T cells in rapamycin and analyzed the FRET signal at selected times after administration. The FRET signal, reflecting the combined effects of surface expression and dimerization, peaked at 8 hours after rapamycin addition, suggesting that DARIC33 activation is time dependent and reaches maximal levels soon after rapamycin addition (Supplemental Figure 8D).

**Figure 7:**
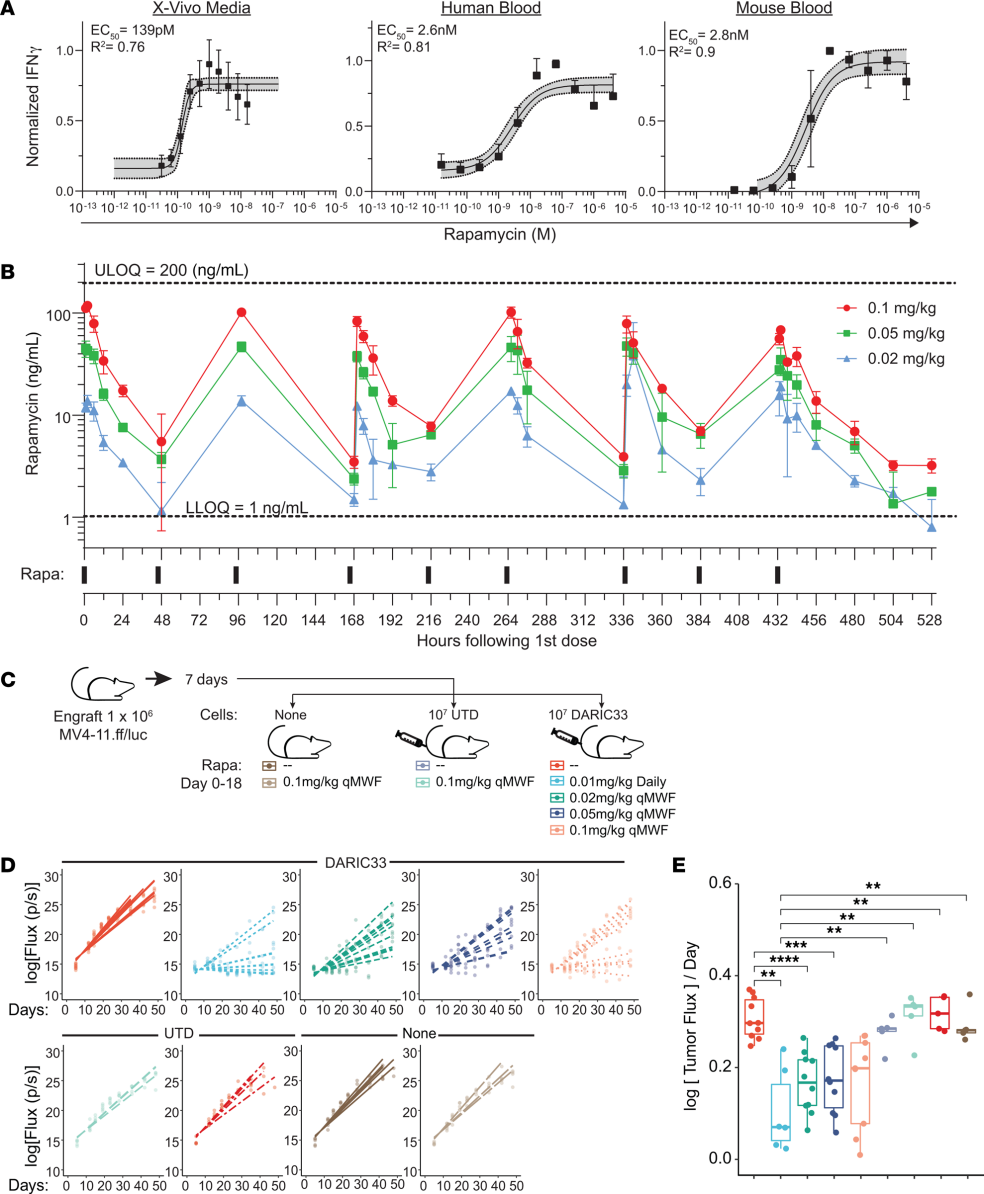
In vitro modeling of SC-DARIC33 rapamycin response allows targeted rapamycin dosing in vivo. (**A**) Cytokine release following stimulation of DARIC33 cells with MV4-11 AML cells in medium or whole blood in the presence of increasing rapamycin concentrations. IFN-γ responses are normalized per donor, and apparent EC_50_ determined using 4-parameter logistic dose-response curves is reported. (**B**) Determination of rapamycin pharmacokinetics in mice. Concentrations of rapamycin in whole blood obtained during administration of various rapamycin doses 3 times weekly are shown above, along with the timing of intraperitoneal rapamycin injections (bars, below). Upper limit of quantitation (ULOQ = 200 ng/mL) and lower limit of quantitation (LLOQ = 1 ng/mL) are indicated. (**C** and **D**) AML tumor progression in mice following treatment with DARIC33 and various dose schedules of rapamycin days 0–18 after T cell infusion. (**C**) Schematic illustrating experimental design. (**D**) Quantitation of tumor growth kinetics. Points represent bioluminescence measures of individual mice (*n* = 5–10 per group), and lines indicate tumor growth trajectories modeled using linear mixed effects. (**E**) Modeled tumor growth rates (slopes of lines in **D**). Points are growth rates modeled for individual mice; boxes and whiskers show mean and SD. **P < 0.01, ***P < 0.001, ****P < 0.0001, 2-tailed *t* tests with Benjamini-Hochberg correction for multiple comparisons.

In patients, rapamycin is highly sequestered by erythrocytes owing to highly abundant cytoplasmic FKBP-related proteins (38) and is bound to plasma proteins (39, 40), which both act to reduce the amount of unbound rapamycin available to bind to DARIC33. We therefore sought to understand the rapamycin concentrations required for DARIC33 heterodimerization and DARIC33 T cell activation in the presence of anticoagulated whole blood using both a FRET-based dimerization assay and an AML-stimulated cytokine release assay, respectively. As with T cell medium (Supplemental Figure 8C), DARIC33 T cells cultured in whole blood exhibited a rapamycin-dependent increase in FRET signal (EC_50_ = 11.4 nM; Supplemental Figure 8E). Next, we performed overnight cocultures of CD33^+^ MV4-11 cells and DARIC33 T cells in either T cell culture medium or human or mouse whole blood samples. The DARIC33 samples exhibited rapamycin-dependent increases in IFN-γ release that were similar in either species (IFN-γ release: human blood, EC_50_ = 2.6 nM; mouse blood, EC_50_ = 2.8 nM; Figure 7A) and among human T cell donors (EC_50_ range of 1.5 nM to 6.3 nM across 3 T cell donors and 2 blood donors, examined in duplicate, *n* = 12 total). The T cell activation assays and FRET dimerization assays showed that higher concentrations of rapamycin (~20-fold and ~100-fold, respectively) are required for half-maximal DARIC33 activity in whole blood as compared with medium. These data define a target range of whole blood rapamycin concentrations capable of activating DARIC33 T cells in the presence of CD33-expressing tumor cells.

We next measured rapamycin exposure following single and repeat intraperitoneal administrations in tumor-bearing mice using a quantitative whole-blood assay. Blood concentrations of rapamycin were generally dose proportional, peaking within 2 hours of administration and decaying with an elimination half-time between 16 and 24 hours (Figure 7B). Peak rapamycin concentrations ranged from 10 ng/mL at doses of 0.02 mg/kg to near 100 ng/mL at a dose of 0.1 mg/kg (Supplemental Table 2).

To determine the impact of various rapamycin dose levels and dosing schedules on the antitumor activity of SC-DARIC33 T cells, we treated MV4-11 tumor–bearing mice with SC-DARIC33 T cells followed by different rapamycin dosing and administration schedules (see Figure 7C and Supplemental Figure 9A for detailed schemata). Among mice receiving dosing regimens predicted to be inactive (e.g., rapamycin alone, UTD T cells with or without rapamycin, or SC-DARIC33 cells alone), tumor growth was similar in comparison with mice receiving no treatment (log[flux]/day = –0.26 to 0.27; Figure 7, D and E). In contrast, treatments predicted to be active (e.g., SC-DARIC33 product followed by rapamycin) exhibited lower rates of tumor growth, with the lowest rate observed among mice receiving 0.01 mg/kg rapamycin intraperitoneally daily (log[flux]/day = 0.058). Tumor growth rates correlated with survival: while control mice developed tumor-associated symptoms near day 45, none of the mice receiving active treatment (DARIC33 plus rapamycin) exhibited signs of tumor progression at this time point. All rapamycin doses tested prolonged survival (log-rank test, *P* < 0.001): at the end of the 90-day observation period, treatment with SC-DARIC33 and 0.01 mg/kg rapamycin daily continued to control tumor outgrowth in 5 of 10 mice (Supplemental Figure 9B).

Interestingly, while in vitro mouse and human whole-blood assays showed similar rapamycin-dependent DARIC33 activation (Figure 7A), we identified species differences in rapamycin red blood cell (RBC) partitioning and plasma protein binding (PPB) (Supplemental Tables 3 and 4). In humans, 94.5% of rapamycin is bound to RBCs while only 3.1% is found in plasma (39). In human plasma, rapamycin is highly protein bound (92%). In contrast, in mice we observed that rapamycin has 5.5% RBC partitioning and is greater than 99% PPB (Supplemental Table 5). Despite these species-specific differences in rapamycin distribution in blood compartments, we observed similar rapamycin EC_50_ for DARIC33 T cell activation in the presence of human or mouse whole blood (Figure 7A), indicating that unbound rapamycin available to interact with DARIC33 was similar in vitro and suggesting that this may also occur in vivo. Taken together, these data support DARIC33 activity across a wide range of rapamycin dosing in vivo and inform a target rapamycin trough (C_24h_) blood concentration range of 1.5–3 ng/mL for DARIC33 T cell activation in humans.

### First-in-human clinical experience demonstrates feasibility of rapamycin activation of SC-DARIC33.

**Figure 8:**
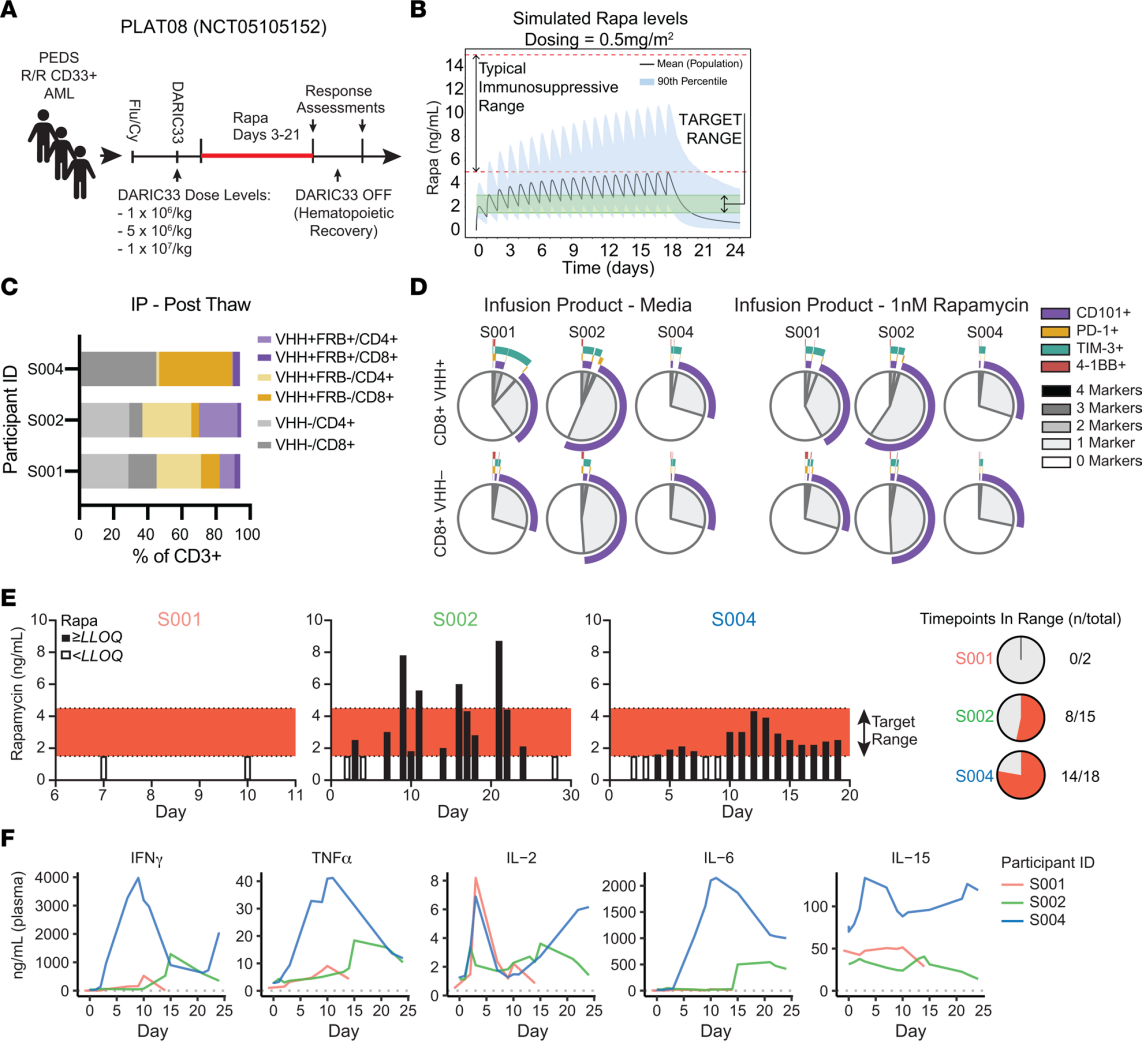
Clinical SC-DARIC33 exhibits activity in patients following accurate targeting of rapamycin levels. (**A**) PLAT-08 clinical treatment schema. After SC-DARIC33 manufacturing, subjects receive lymphodepleting fludarabine and cyclophosphamide (Flu/Cy) and SC-DARIC33 at 1 of 3 assigned dose levels on day 0. Rapamycin is administered on days 3–21. Bone marrow biopsies are conducted for response assessments on days 21 and 28. (**B**) Simulated serum rapamycin concentrations using population pharmacokinetic modeling. Daily administration of 0.5 mg/m^2^ rapamycin achieves trough concentrations above the target range for SC-DARIC33 activation and peak concentrations below immunosuppressive doses of rapamycin for most pediatric subjects. (**C**) Characteristics of thawed clinical SC-DARIC33 cell products administered to trial participants. The proportion of cells expressing surface DARIC components as assessed by flow cytometry is shown. (**D**) Expression of activation markers by clinical infusion cell products following overnight culture in medium alone or medium supplemented with 1 nM rapamycin. (**E**) Frequent reevaluation enables successful targeting of serum rapamycin levels in patients. The proportion of time points (both peak and trough levels) within the target range (1.5–4 ng/mL) is shown at right. (**F**) Elevation of serum cytokines associated with T cell activation is observed following administration of SC-DARIC33. Traces show cytokine levels for samples obtained from each patient. Values reported are the mean of *n* = 2 replicates.

We designed PLAT-08 (NCT05105152, ClinicalTrials.gov) as a first-in-human phase I trial evaluating the safety of escalating doses of SC-DARIC33 in pediatric and young adult patients with relapsed and refractory AML (Figure 8A). In this trial, subjects receive lymphodepleting chemotherapy followed by SC-DARIC33 T cell products and rapamycin. To identify rapamycin doses and schedules that maximize the likelihood of achieving rapamycin blood concentration troughs within the target range of 1.5–4 ng/mL, we simulated rapamycin dose-exposure relationships from adult and pediatric patients by using population pharmacokinetic models (38, 41) and sampled anthropomorphic measurements for children (Supplemental Figure 10, A and B). Among evaluated dosing schedules (Supplemental Figure 10, C–H), rapamycin daily dosing of 0.50 mg/m^2^ (for patients ≤1.5 m^2^) or 0.75 mg (for patients >1.5 m^2^) is predicted to achieve target rapamycin trough concentrations above 1.5 ng/mL and peak concentrations below 8 ng/mL in 90% of the pediatric population (Figure 8B). The mean population rapamycin peak and trough (C_24h_) levels are predicted to be between 2 ng/mL and 4 ng/mL, well below the range of rapamycin typically used for immunosuppression in solid organ transplant recipients (12–24 ng/mL; ref. 42). We therefore selected the dose schedule of daily oral rapamycin at 0.5 mg/m^2^ for initial evaluation in pediatric patients.

To evaluate whether rapamycin exposure is associated with evidence of SC-DARIC33 tumor reactivity in patients, we evaluated infusion products and blood samples obtained from the first 3 patients enrolled on PLAT-08 and treated at dose level 1 (DL1 = 10^6^ DARIC^+^ cells per kg). Infusion products contained between 49% and 57.9% DARIC^+^ T cells (defined as those expressing surface V_H_H). We developed a high-dimensional flow cytometry panel capable of simultaneously evaluating markers of myeloid and T cell identity (CD33, CD3, CD4, and CD8), lentiviral transduction (V_H_H and FRB), and T cell activation state (CD101, PD-1, and 4-1BB) in patient samples. We found that similar proportions of DARIC^+^ and DARIC^-^ populations among patient infusion products (Figure 8C). We also found similar proportions of CD8^+^ T cells expressed of activation markers following incubation of patient infusion products in medium alone or medium supplemented with rapamycin, demonstrating that exposure to rapamycin alone was insufficient to stimulate T cell activation (Figure 8D). Using continuous rapamycin monitoring and dose adjustment, rapamycin concentrations in blood samples among the 3 subjects (S001, S002, and S004) were within the target range in 0 of 2 time points, 8 of 15 time points, and 14 of 18 time points, respectively (Figure 8E). Finally, we monitored temporal trends in serum levels of a broad panel of cytokines analytes selected for associated with CAR T cell activation (34, 35) and successful CAR T cell therapy of lymphocytic leukemia (43). Serum samples from patient S004, which had the highest proportion of rapamycin concentration in blood falling within the target range, exhibited dramatic increases in IFN-γ, TNF-α, and IL-6, peaking around day 10 after CAR infusion, followed by steady declines (Figure 8F). These initial observations indicate that successfully achieving the target rapamycin concentration is associated with elevated levels of cytokines affiliated with CAR T cell activation.

**Figure 9:**
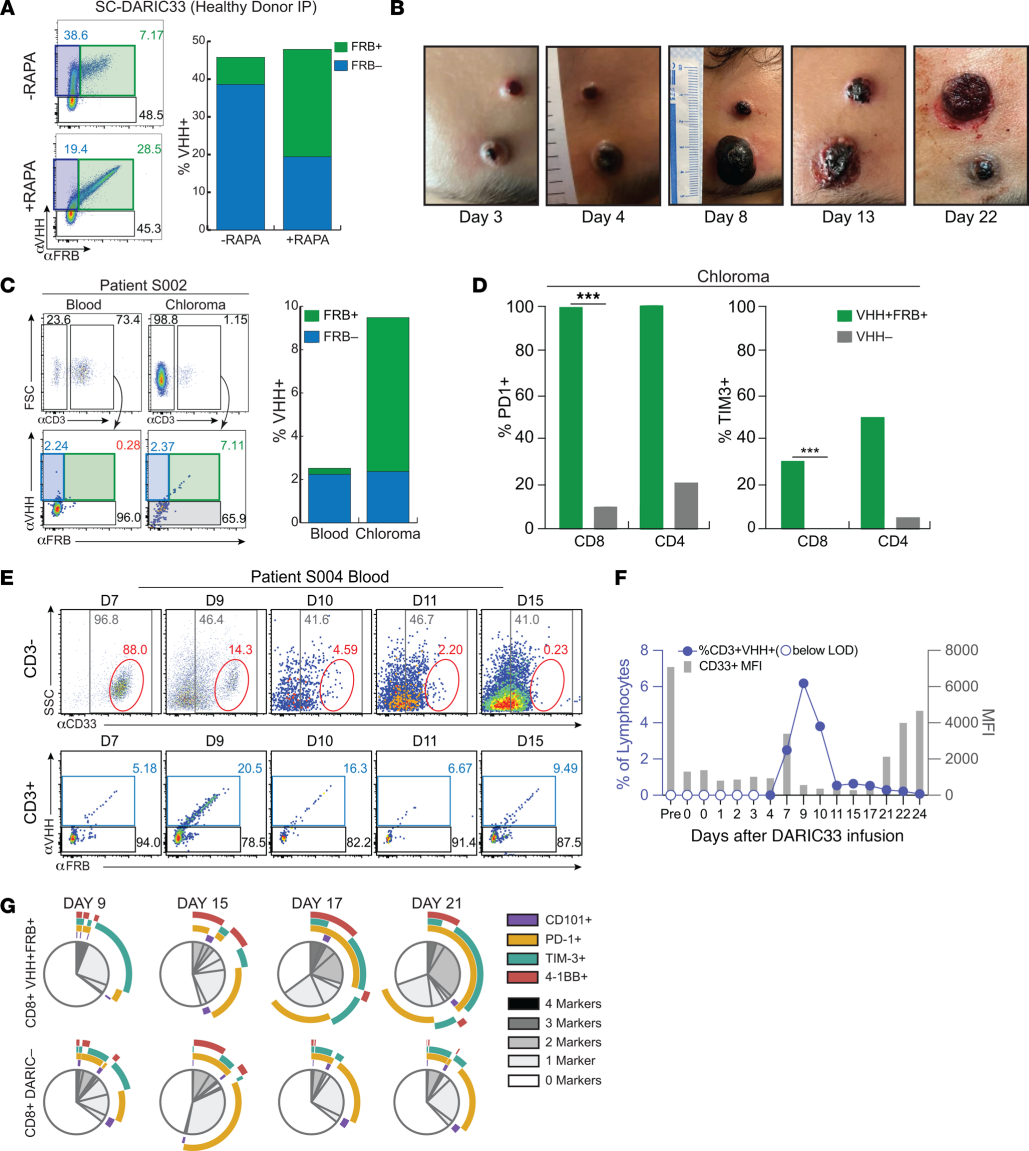
Clinical activity of rapamycin-activated SC-DARIC33 in patients. (**A**) Expression of FRB by SC-DARIC33 is correlated with rapamycin exposure. SC-DARIC33 manufactured from a healthy donor was cultured overnight in medium alone or media supplemented with 1 nM rapamycin. The proportion of V_H_H^+^ and FRB^+^ cells is shown in the bar graph. Note the rightward shift of V_H_H^+^ cells following rapamycin exposure. (**B**) Progressive inflammatory changes and hemorrhagic conversion of a chloroma following administration of SC-DARIC33 to subject S002. Samples from chloroma tissues are shown in **C** and **D**. Photographs used with permission. (**C**) Rapamycin-activated FRB^+^ DARIC33 T cells are expanded within chloroma tissue. Paired blood and chloroma tissue from patient S002 were evaluated by flow cytometry. T cells expressing CD3 were analyzed for V_H_H and FRB expression. The proportion of V_H_H^+^ and FRB^+^ cells among CD3^+^ cells is shown in the bar graph. (**D**) Rapamycin-activated DARIC33 cells within chloroma tissue obtained from patient S002 express increased markers of activation including PD-1 and TIM3. The proportion of either V_H_H^+^FRB^+^ cells (green bars) or V_H_H^–^ cells (gray bars) expressing PD-1 or TIM3 is shown. ***P < 0.001, χ^2^ test with Bonferroni correction for multiple tests. (**E**) Peripheral blood from patient S004 shows concurrent expansion of DARIC33 cells and reduction of CD33^hi^ cells. (**F**) Quantification of antigen abundance, as measured by MFI, and expansion of SC-DARIC33 cells within blood samples. Peak SC-DARIC33 expansion is followed by decreased CD33 antigen expression. (**G**) Expression of activation/exhaustion markers by rapamycin-activated SC-DARIC33 cells, as assessed by flow cytometry. Boolean gating results are shown as pie graphs with overlapping arcs indicating multi-antigen expression. At later time points (days 17 and 21), expression of activation markers is increased among V_H_H^+^FRB^+^ cells.

Following observations that rapamycin stabilized surface FRB on DARIC33 cells (Figure 1E), we hypothesized that FRB expression would correlate with rapamycin exposure. As expected, evaluation of healthy donor T cell products using our clinical flow cytometry panel showed that overnight exposure to rapamycin resulted in increased V_H_H and FRB expression (Figure 9A). Patient S002 exhibited chloromas, some of which developed increased hemorrhagic necrosis following SC-DARIC33 and rapamycin administration (Figure 9B). Flow cytometric evaluation of chloroma and peripheral blood tissue demonstrated preferential accumulation of V_H_H^+^FRB^+^ cells within the chloroma tissue (Figure 9C). These cells had an activated phenotype, as the proportion of PD-1^+^ and TIM3^+^ cells was higher among the V_H_H^+^FRB^+^ CD8^+^ T cells compared with V_H_H (DARIC33) CD8^+^ T cells (Figure 9D). We next analyzed T cell expansion and functionality in patient S004. Among peripheral blood samples from patient S004, the proportion of circulating blast-like CD33^hi^ side scatter–low (SSC^lo^) cells decreased by 99.8%, from 88% to 0.23%, from day 7 to day 15 (Figure 9E). This was accompanied by concurrent expansion of DARIC33^+^ T cells within the peripheral blood, peaking at 6% of total lymphocytes and 20.5% of circulating T cells on day 9 after CAR infusion before contracting (Figure 9F). Evaluation of surface phenotypes of V_H_H^+^FRB^+^ (RAPA-exposed) CD8^+^ T cells showed progressively increasing expression of activation markers, including PD-1, TIM3, and 4-1BB, within RAPA-exposed CD8^+^ T cells from day 9 through day 21 (Figure 9G). In contrast, DARIC33-negative CD8^+^ T cells presented with a transient increase in surface TIM3 and PD-1 at day 15 that was not sustained. These data show that, in the presence of tumor antigen and rapamycin, SC-DARIC33 expands, engrafts, and acquires activated states. SC-DARIC33 activation and expansion were temporally coincident with increases in cytokine markers of T cell activation and transient depletion of CD33^hi^ cells in peripheral blood. Together, these findings provide initial in-human evidence that the DARIC33 platform achieves rapamycin-responsive antigen-dependent T cell activation.

## Discussion

In this report we describe preclinical characterization and first clinical data from a distinct CAR T cell platform that aims to solve difficult challenges in treating AML through a drug-regulated DARIC architecture. Targeting AML with CAR T cells presents specific challenges, as overlapping expression of target antigens on myeloid cells and hematopoietic stem cells limits the therapeutic window for constitutively active CARs. Here, we describe the development of a regulated anti-AML CAR T cell therapy that targets a membrane-proximal domain CD33 epitope. Rapamycin-dependent heterodimerization of DARIC components results in a stringent off state in the absence of rapamycin and acquisition of an effector on-state T cell in the presence of low-nanomolar rapamycin concentrations. As a benchmark, we compared DARIC33 T cells with a CD19 CAR production design that has achieved clinical efficacy (34, 35). The two architectures demonstrate similar potency in vitro and comparable potency in challenging in vivo models. The DARIC33 system, composed exclusively of human or humanized domains, and using clinically tolerable dosing of an FDA-approved drug (rapamycin), represents a substantial advance over other regulated CAR formats. We have initiated clinical testing of the DARIC33 system and observed endpoints consistent with T cell activation, expansion, and early signs of antitumor activity.

Precise control of CAR T cell activity may help mitigate toxicities associated with CAR T engraftment syndromes such as cytokine storm and/or the aplasia that occurs due to targeting of a cell lineage–specific antigen such as CD19 or CD33. After infusion, synchronous CAR T cell activation by abundant tumor cells or their nonmalignat antigen-expressing counterparts can stimulate dramatic proliferation and release of effector cytokines, resulting in potentially fatal cytokine release syndrome and neurotoxicity (2, 44). These adverse effects limit CAR dosing but could potentially be mitigated by pausing of CAR activity such that DARIC products infused in the off state may be subject to pulses of rapamycin induction to drive engraftment and incrementally reduce tumor burden. While the risks of B cell aplasia following CD19 CAR T cell therapy may be mitigated by immunoglobulin infusions, indefinite elimination of cells expressing AML-associated antigens such as CD33, CD123, CLL1/CLEC12A, or CD38 is likely to result in clinically intolerable myelosuppression. Thus, when treating AML, strategies to mitigate hematopoietic toxicity are likely to be a requirement.

Individualizing off-state on-state sequencing may tailor therapeutic windows to patient-specific circumstances (45) and represents a useful feature of the DARIC platform. Intermittent cycling of DARIC33 activity through metronomic rapamycin dosing may enable episodic hematopoietic recovery between cycles of active leukemic targeting. While the half-life of rapamycin precludes rapid cessation of DARIC T cell function, pharmacologic inhibitors of proximal antigen receptor signaling, such as dasatinib (46, 47), in combination with rapamycin withdrawal may represent an alternative strategy for managing acute toxicities arising from unrestrained T cell activation. In addition, periods of alternating signaling and quiescence may enhance the efficacy of antitumor T cells by preventing T cell exhaustion (48–50) and allowing effector T cells to transition to memory states (29, 30, 51–54) after periods of prolonged antigen exposure. We are currently evaluating transcriptional and epigenetic changes in SC-DARIC33 cells following rapamycin interval dosing to analyze the impact of paused T cell activity on T cell memory state transitions. Temporal pauses of CAR activity may therefore be a general method to promote or sustain the fitness of engineered therapeutic T cells.

Controllable CAR designs may open new paradigms of CAR T cell therapy that directly address both prevailing failure mechanisms and risks to patients. For example, controllable CAR T cells may allow administration of higher cell doses followed by individualized titration of the activating drugs, widening therapeutic windows (45). In addition, whereas constitutive potency-enhanced CARs risk runaway reactivity that may be difficult to bring back under control (55, 56), regulated CAR designs may promote the safety of genetic potency enhancement strategies that attempt to further CAR T cell survival, expansion, or reactivity. Finally, if intermittent T cell activation leads to the formation of a long-lived DARIC T cell niche, patients could be redosed with rapamycin to control any tumor recurrence following the initial remission. Overall, clinical validation of a controllable CAR T cell design will impact multiple research questions and clinical outcomes within the cellular therapy field. The ongoing first-in-human trial of SC-DARIC33 for children and young adults with relapsed or refractory CD33^+^ AML will provide clinical and correlative data supporting the pharmacologic control of CAR T products, as well as AML- and myeloid cell–targeting attributes of this next-generation cellular therapeutic.

## Methods

### Sex as a biological variant.

Murine xenograft studies used female mice to minimize size variation. Results are expected to be relevant to all humans.

### Cell lines.

Cell lines were obtained from the following sources: MOLM14 (ACC 777) and MV4-11 (ACC 102) were purchased from the Leibniz Institute DSMZ; A549 (CCL-185), THP1 (TIB-202), and HL-60 (CCL-24) were purchased from ATCC; HL-60 was provided by the Bhatia lab (Fred Hutchinson Cancer Center). HL-60, MV4-11, and MOLM14 were engineered to express GFP and firefly luciferase via lentiviral transduction. GFP^+^ cells were FACS sorted to generate uniformly positive cell population. Raji cells engineered to express GFP and firefly luciferase were further modified to express CD33M (NM_001772) by lentiviral transduction, followed by FACS sorting and limiting dilution cloning to select expression of equivalent CD19 and CD33 antigen concentrations. K562 was transduced with lentivirus to express membrane-bound OKT3 as a positive control for in vitro T cell activity assays. HL-60, MOLM14, MV4-11, and THP1 cells were cultured in RPMI 1640 medium supplemented with 10% FBS and 1% l-glutamine, referred to as complete RPMI.

### CD33-targeted DARIC-V_H_H lentiviral vector design and production.

DARIC lentiviral vectors were generated as previously described (4). Briefly, transgenes encoding the CD33-specific V_H_H binders were synthesized incorporating sequence modifications that optimized codon usage and enhanced human immune tolerance and cloned into the previously described CD19-DARIC transfer plasmid (4) using Gibson cloning (New England Biolabs). Cloned products were verified using Sanger sequencing.

A 4-plasmid self-inactivating lentiviral production system was used. Briefly, the DARIC transfer vectors mixed with envelope and packaging vectors were transfected into 293T cells using TransIt transfection reagent (Mirus Bio). Vector-containing supernatant was collected, passed through a 0.2 μm filter, and either used immediately or stored at –80°C until use. In some cases, vector supernatant was concentrated by centrifugation at 10,000*g* for 4 hours before cryopreservation. Analysis of vector copy number was performed as described previously (4).

### DARIC T cell manufacture.

Thawed PBMCs were resuspended in TCGM (T cell growth media) composed of X-VIVO 15 (Lonza) supplemented with 10mM HEPS and 2mM Glutamax (both from ThermoFisher Scientific), 5% human AB serum (Valley Biomedical) and supplemented with 250 IU/mL recombinant human IL-2 (catalog 78220.3, Stemcell) before activation with 50 ng/mL anti-CD3 (clone OKT3) and anti-CD28 (clone 15E8) antibodies (Miltenyi Biotec). Lentivirus supernatants were added to PBMC cultures 24 hours later (multiplicity of infection = 10). Seventy-two hours after activation, transduced PBMCs were collected, washed, and resuspended in complete TCGM with human IL-2 at 0.5 × 10^6^ cells/mL and transferred to gas-permeable culture vessels (G-Rex, Wilson Wolf). PBMC cultures were expanded in vitro at cell density of 0.5 × 10^6^ to 2 × 10^6^ cells/mL maintained by the addition of fresh medium every 2–3 days for a total of 10–11 days until cryopreservation. Clinical T cell product manufacture was completed essentially as described previously (57), except T cell cultures were initiated with a 1:1 ratio of CD4^+^ and CD8^+^ T cells.

### Cytokine release assay.

For cytokine production analysis, 0.1 × 10^6^ T cells were cocultured with 5 × 10^4^ target cells (effector/target ratio = 2:1) for 24 hours with or without rapamycin (1 nM, unless otherwise specified) in TCGM. Culture supernatants were evaluated using the V-PLEX Proinflammatory Panel 1 Human kit (Meso Scale Diagnostics) and analyzed by the MESO QuickPlex SQ 120 Instrument (Meso Scale Diagnostics) according to the manufacturer’s instructions.

### Murine xenograft models.

Female adult (8- to 12-week-old) NOD/scid IL-2RC^null^ (NSG) mice were bred in-house or purchased from The Jackson Laboratory, housed in specific pathogen–free conditions with a 12-hour light/12-hour dark cycle, and monitored daily by veterinary staff or research scientists. All experiments were carried out following Institutional Animal Care and Use Committee–approved protocols. Mice exhibiting hunched posture, decreased mobility that impaired feeding, single or multiple tumors totaling more than 1 cm in diameter, more than 20% weight loss, or loss of skin integrity were humanely euthanized. Development of tumor-associated symptoms requiring euthanasia was considered an event for the purposes of Kaplan-Meier analyses.

Cultures of tumor cells modified for bioluminescence imaging (BLI) were washed and resuspended in PBS. Cell densities were adjusted to contain the following cell doses within 200 μL as follows: HL-60, 5 × 10^6^; MV4-11, 1 × 10^6^; MOLM14, 1 × 10^5^; or Raji, 0.5 × 10^6^. Tumor cell suspensions were administered via lateral tail vein injection.

BLI was performed by intraperitoneal or subcutaneous injection of 4.29 mg per mouse of d-luciferin (Xenogen) at various time points prior to and after tumor inoculation. Before treatment, mice were distributed so that treatment groups had similar median bioluminescence. Mice exhibiting tumor signal only within the tail were excluded from studies. Imaging of isoflurane-anesthetized mice occurred 15 minutes after d-luciferin injection using the IVIS Spectrum Imaging System (PerkinElmer). Luciferase activity was analyzed using Living Image Software version 4.5.2 (PerkinElmer).

Before administration to recipient mice, cryopreserved T cell products were thawed into human AB serum, washed with PBS, counted, and resuspended in PBS such that a single dose of T cells was administered in a total volume of 200 μL. Cell suspensions were maintained on ice until injection via the lateral tail vein of recipient mice. Mice received a single injection of cells. To determine cell dosing, the total number of DARIC33 T cells and CD19-DARIC T cells administered to mice was calculated on the basis of FRB^+^ cells, i.e., total cells *=* desired cell dose / (proportion FRB^+^). Similarly, the total number of CD19 CAR T cell products was determined on the basis of the proportion of cells expressing the EGFRt marker (58). The total cell dose of untransduced (UTD) control T cell products was matched to highest total number of T cells administered within a given experiment.

Mice assigned to rapamycin treatment received rapamycin by intraperitoneal injection either daily or every Monday, Wednesday, and Friday as specified within schemata and/or figure legends. Solutions of rapamycin for injection were prepared by dilution of a 10 mM DMSO stock into PBS immediately prior to administration such that final concentration of DMSO was less than 0.2% (vol/vol). For weight-based dosing, mouse weights were determined weekly and used to adjust rapamycin dosing. The total volume of rapamycin solution administered ranged from 50 μL to 200 μL.

### Evaluation of blood, serum, and chloroma samples from patients.

After enrollment, CD4^+^ and CD8^+^ T cells isolated from cells collected by leukapheresis were combined in a 1:1 ratio to manufacture SC-DARIC33 as previously described for CD19 CAR T cells (57). Freshly obtained blood, marrow, or chloroma samples from patients after SC-DARIC33 infusion were evaluated by immunophenotyping following RBC lysis using standard staining and flow cytometry techniques (see Supplemental Methods for additional details).

### Statistics.

Statistical significance was determined by a *P* value of less than 0.05 using GraphPad Prism 9 software or the lme4 package of the R statistical computing package. Tumor symptom–free survival of mice within studies was compared using Kaplan-Meier method and the log-rank test. Global comparisons for studies with more than 2 groups were conducted, and if significant, pairwise comparisons were examined using a FDR of 0.05. Logistic dose-response curves were evaluated in GraphPad Prism 9.

### Study approval.

PLAT-08 is an ongoing phase I study of CD4^+^ and CD8^+^ T cells lentivirally transduced to express the DARIC33 transgene, delivered via intravenous infusion following lymphodepleting chemotherapy in pediatric and young adult patients (< 30 years old) with relapsed or refractory acute myeloid leukemia (NCT05105152). The study is conducted in accordance with FDA and International Conference on Harmonization guidelines for good clinical practice, the Declaration of Helsinki and applicable institutional review board guidelines (study protocol approved by Seattle Children’s Hospital Institutional Review Board). All patients or their guardians provided written informed consent for trial participation. Written informed consent was received for the use of photographs, and the record of informed consent has been retained at Seattle Children’s.

### Data availability.

Requests for materials should be directed to the corresponding authors and will be fulfilled upon completion of appropriate material transfer agreements. High-throughput sequencing data (RNA-Seq) were deposited in the NCBI’s Gene Expression Omnibus database (GEO GSE255002). Code used to evaluate CD33 splicing from sequence read archives will be made available upon request to AA. Supporting Data Values associated with each figure are provided in a supplemental spreadsheet.

See Supplemental Methods for additional study details and descriptions.

## Author contributions

JA, AEP, KO, GT, MF, JJ, MP, JAG, AA, and MCJ conceptualized the project. JA, AEP, KO, GT, MF, ML, DEZ, SRR, NT, SKH, MP, JAG, and AA developed the methodology. JA, KO, JZ, WHL, UM, ARK, DX, PPLS, SKH, CE, SS, RL, PL, MF, NN, SWF, AL, RAG, SS, KJ, AS, WC, JT, AH, BE, SB, JW, SRR, and NT conducted the investigation. JA, AA, SRR, NT, and DEZ were responsible for visualization. JA, MCJ, JJ, and PDG acquired funding. JA, JJ, JAG, and MCJ were responsible for project administration. JA, JJ, JAG, AA, and MCJ supervised the project. JA and AA wrote the original draft of the manuscript. JA, JJ, MP, PDG, JAG, AA, and MCJ reviewed and edited the manuscript.

## Supplementary Material

Supplemental data

Supporting data values

## Version 1 03/19/2024

In-Press Preview

## Version 2 05/01/2024

Electronic publication
