# Supplementary material for: Drug-regulated CD33-targeted CAR T cells control AML using clinically optimized rapamycin dosing
Source: J Clin Invest. 2024 Mar 19;134(9):e162593. doi: 10.1172/JCI162593 (PMC11060733; doi:10.1172/JCI162593)
Supplement: Supplemental data [file jci-134-162593-s222.pdf]

Drug-regulated CD33-targeted CAR T cells control AML using clinically optimized rapamycin dosing

Authors: Jacob Appelbaum[1,2,3,4*], April E. Price[5], Kaori Oda[1], Joy Zhang[5], Wai-Hang Leung[5], Giacomo Tampella[1], Dong Xia[5], Pauline P. L. So[5], Sarah K. Hilton[5], Claudya Evandy[1], Semanti Sarkar[1], Unja Martin[5], Anne-Rachel Krostag[5], Marissa Leonardi[1], Daniel E. Zak[5], Rachael Logan[1], Paula Lewis[5], Secil Franke-Welch[5], Njabulo Ngwenyama[5], Michael Fitzgerald[1], Niklas Tulberg[1], Stephanie Rawlings-Rhea[1], Rebecca A. Gardner[1], Kyle Jones[6], Angelica Sanabria[6], William Crago[6], John Timmer[6], Andrew Hollands[6], Brendan Eckelman[6], Sanela Bilic[7], Jim Woodworth[7], Adam Lamble[1,4], Philip D. Gregory[5], Jordan Jarjour[5], Mark Pogson[5], Joshua A. Gustafson[1], Alexander Astrakhan[5*], Michael C. Jensen[1*]

Supplementary Materials

Materials and Methods 3
- Reagents 3
- Deletion of CD33 via genome editing 3
- Single Domain Camelid Antibody Screening 4
- Production and characterization of recombinant CD33-binding VHH proteins 4
- Primary Human Cell Isolation 5
- Spheroid cytotoxicity analysis 5
- Assessment of Hematopoietic Colony Formation after CAR Exposure 6
- RNA sequencing analysis of DARIC33 T cells 6
- Kinetic assessments of DARIC33 activity after rapamycin removal 7
- Clinically Appropriate DARIC33 Cell Manufacture 8
- Assessment of DARIC33 activity in whole blood 8
- Measurement of rapamycin concentrations in mouse blood 9
- Assessment of red blood cell partitioning of rapamycin in mouse blood 9
- Assessment of plasma protein binding of rapamycin in mouse and human serum 11

Pediatric Rapamycin Exposure Modeling 13
Flow cytometry 14
Fluorescence resonance energy transfer (FRET) analysis of DARIC33 dimerization 14
Quantification of CD33 antigen density 15
Analysis of CD33m expression in AML transcriptomes and healthy tissues 15
Rapamycin Population Pharmacokinetic Modeling 17
Evaluation of patient blood, serum and chloroma samples 18
Processing of patient biofluids 18
Evaluation of patient serum using electrochemiluminescence assays 18
Study Approval 19
Statistical Analysis 20
Additional References 20
Supplementary Figures 23
Supplemental Figure 1: CD33-specific VHH exhibit a range of affinities towards CD33 protein and produce functional DARIC33 lentiviral vectors. 23
Supplemental Figure 2: DARIC33 is highly responsive to both antigen expression and rapamycin dosing even in the presence of soluble antigen 25
Supplemental Figure 3: DARIC33 T cells are specific for CD33 and can modulate growth of an antigen-low tumor model. 27
Supplemental Figure 4: A single V_HH binder is specific for the short CD33m isoform, which is broadly expressed in AML samples 29
Supplemental Figure 5: DARIC33 T cells exhibit a transcriptional expression profile specific to the presence of both antigen and rapamycin. 31
Supplemental Figure 6: DARIC33 T cells had similar functional activity and lower tonic signaling compared to CAR33 T cells 33
Supplemental Figure 7: DARIC33 T cells drive cytotoxicity in a cell dose dependent manner. 35
Supplemental Figure 8: FRET analysis demonstrates DARIC33 dimerization in the presence of rapamycin 36
Supplemental Figure 9: DARIC33 T cells control tumor growth in vivo over a wide range of rapamycin concentration 38
Supplemental Figure 10: Integration of preclinical models and simulated rapamycin dose exposure relationships reveals dosing strategy predicted to activate DARIC33 in vivo without reaching immunosuppressive concentrations. 39
Additional Supplementary Data Items: 41
Supplemental Table 1: Quantification of endogenous and engineered CD33 expression on various cell lines. Cell lines in bold were used to test T cell functionality 41

Supplemental Table 2: Rapamycin half-life in NSG mice as a function of input dosing......... 42
Supplemental Table 3: Red Blood Cell Partitioning of Rapamycin in NSG Mouse Whole Blood (K_2EDTA) 42
Supplemental Table 4: Rapamycin (2mM) Protein Binding in CD-1 mice, NSG mice, and Human Plasma (K_2EDTA).. 42
Supplemental Table 5: Rapamycin (2mM) Protein Binding in 10% NSG Mouse Plasma (K_2EDTA).. 43

MATERIALS AND METHODS

Reagents

Rapamycin solution was purchased from Selleckchem (catalog S1039) and stored in single use aliquots at -20°C. Frozen aliquots were diluted in PBS immediately prior to in vitro or in vivo use. RPMI-1640 (catalog 22400-089), HEPES (catalog 15630-080), GlutaMax (catalog 35050-061) and L-glutamine (catalog 25030-081) were purchased from Thermo Fisher Scientific and Fetal Bovine Serum (FBS) was purchased from VWR (catalog 97068-085). Human AB sera were from Valley Biomedical. X-VIVO 15 (BE08-959H) media were purchased from Lonza. T cell growth medium (TCGM) was prepared with X-VIVO 15 supplemented with 10mM HEPES, 2mM GlutaMax, and 5% human AB serum.

Deletion of CD33 via genome editing

To generate CD33-deficient cell lines, a CD33-targeting CRISPR guide oligonucleotide (CCTCACTAGACTTGACCCAC, Synthego) was and complexed with Cas9 protein to generate RNPs. The HL60 and THP1 cell lines were electroporated with RNPs using the 4D-Nuclecofactor instrument using manufacture’s recommendations (Lonza). Electroporated cells were returned to culture, expanded, and sorted by flow cytometry to enrich for loss of CD33 expression. Knockout efficiency was analyzed by staining with an anti-CD33 antibody (Clone P67.7) and via genomic analysis[1].

Sorted CD33 knockout cell lines were subject to limiting dilution to obtain single clones. Wells containing single clones were expanded and loss of expression was confirmed by molecular, flow cytometric and functional analyses. The derived monoclonal knockout lines were used for in vivo xenograft studies.

Single Domain Camelid Antibody Screening

Alpacas and llamas were immunized and boosted with recombinant CD33. Specific antibody titers were determined by flow cytometry on CD33 positive and negative cells, using a dilution of serum from immunized animals and Alexa Fluor 647 conjugated protein A (Invitrogen). Subsequently, PBMCs were isolated from whole blood of immunized animals using ficoll gradient centrifugation. RNA was isolated by RNeasy midi kit (Qiagen) and first strand cDNA was generated via SuperScript IV Reverse Transcriptase (Invitrogen). VHH yeast display libraries were generated via specific amplification of the heavy chain variable domain of the IgG2 and IgG3 subclasses and screened by flow cytometry for binding to recombinant human CD33 and the splice variant CD33m. Lead VHHs sequences were recovered by sequencing yeast colonies.

Production and characterization of recombinant CD33-binding VHH proteins

VHH-Fcs were transiently expressed in Expi-CHO cells (Thermo Fisher) and purified by protein A chromatography. Binding assessments were conducted using CHO cells transiently transfected with plasmids encoding either CD33M or CD33m, fused in-frame with the GFP gene. Antibodies were titrated 1:3 starting at 1uM and detected using an AF-647 conjugated anti-human Fc secondary (Jackson Immunoresearch) and read on a IQue flow cytometer (Sartorius). Cells expressing the CD33 variants were identified via gating on the GFP positive populations.

Binding characterization was evaluated using surface plasmon resonance (SPR) on a Biacore T200 instrument. Purified VHH1-Fc was captured on an anti-human IgG immobilized sensor surface. A recombinant CD33-His titration series (0, 0.16, 0.8, 4, 20 and 100nM) was injected using the single cycle kinetics method. Association time was set to 120 seconds and dissociation time was set to 300 seconds. Parameters for binding kinetics were fit using a 1:1 binding model.

Primary Human Cell Isolation

Primary human peripheral blood mononuclear cells from healthy donors and obtained via an IRB exempt protocol were isolated from discarded LRS cones (BloodworksNW) using density centrifugation (Lymphoprep, Stemcell). Primary human CD4+ and CD8+ T cells were isolated from apheresis products supplied from healthy donors (BloodworksNW) using paramagnetic beads (StraightFrom Leukopack CD4/CD8 Microbead Kit, Human, Miltenyi Biotec) and the MM24 instrument (Miltenyi) according to the manufacturer's instructions. Isolated cell fractions were frozen in CryoStor CS5 cryopreservation media and stored in vapor phase liquid nitrogen until use.

Spheroid cytotoxicity analysis

A549 tumor cells engineered to express CD33 and NucLight Red (Sartorius) via lentiviral transduction were plated into 96 well ultra-low attachment plates (Corning) and cultured for 3 days to allow for spheroid formation. T cells were then added to spheroids and cytotoxicity was assessed by quantifying loss of red fluorescence using an Incucyte instrument and software (Sartorius).

Assessment of Hematopoietic Colony Formation after CAR Exposure

T cells were co-cultured for 4 hours at a 10:1 effector to target ratio with thawed cryopreserved CD34+ hematopoietic stem and progenitor cells in the presence or absence of 1nM rapamycin. Following incubation, the cell mixture was plated in semi-solid MethoCult media (Stem Cell Technologies) to allow for hematopoietic colony formation. Colonies of defined morphology were enumerated 14 days later using a light microscope.

RNA sequencing analysis of DARIC33 T cells

DARIC33 T cells generated from four donor PBMC samples were cultured with media or media supplemented with 1 nM rapamycin for 24 hours, washed and then stimulated with MV4-11 tumor cells or left unstimulated for an additional 24 hours. Cells were then sorted into CD4+ and CD8+ populations using a FACSAria (BD Biosciences), giving 32 samples altogether. Total RNA was isolated (Qiagen 96-well RNeasy Prep Kit) and used to generate libraries using KAPA Hyperprep Kit with RiboErase and KAPA Unique Dual-Indexed Adapter Kit (Roche). Samples were sequenced on a NextSeq500 instrument with paired-end 150bp reads. Reads were filtered for quality and aligned to the Genome Resource Consortium hg38 human genome reference[2] using STAR[3]. We removed one sample with poor read quality (mapped reads < 80%). Gene expression was quantified as transcripts per million (TPM) using RSEM[4]. Gene names were harmonized using the HUGO Gene Nomenclature Committee (HGNC, www.genenames.org) and genes with a maximum TPM of 0 within a cell population were removed.

Analysis of differential expression in the response to antigen stimulation in the context of rapamycin was focused on 2,792 genes that were previously associated with CAR T function and/or encode putative cell surface proteins. This gene set is the union of genes from the NanoString CAR T characterization panel (*LBL-10664-01_CAR-T_Characterization_Gene_List.xlsx*,

NanoString Inc.), the BioLegend Legend Screen (LEGENDScreen_Human_PE_Kit_Specificity_List_R2_1.xlsx, BioLegend, Inc.), and the *in silico surfaceome* reported by Bausch-Fluck et al.[5].

Differential expression of a subset of genes informative of CAR T cell states (see supplemental file) in the “DARIC active” condition was tested using a linear mixed model fit by the statsmodels python package[6] on a gene-by-gene basis for the CD4 and CD8 populations separately. The form of the model is

$$y_i = \beta_{0,\mathrm{i}} + \beta_{RAPA,i} + \beta_{Ag,i} + \beta_{RAPA\bullet Ag,i} + \theta_{D,i} + \theta_i$$

where y_i is the log2(TPM + 0.001) for gene *i*; $\beta_{RAPA,i}$ and $\beta_{Ag,i}$ are fixed effects on gene *i* of rapamycin or antigen treatment; $\beta_{RAPA:Ag,i}$ is the fixed effect on gene i of interactions between rapamycin and antigen; $\theta_{D,i}$ is the random effect of Donor on gene *i*; and $\beta_{0,i}$ and θ_i are intercept and residual errors, respectively. Significant differential expression in the “DARIC active” condition was defined as $p \leq 0.05$ for the interaction term $\beta_{RAPA:Ag,i}$ after correcting for multiple hypothesis comparisons using the Benjamini-Hochberg method. This parameter specifically captures gene expression changes that occur only when both rapamycin and antigen are present.Enrichment of T cell activation markers in the significant genes identified was tested using a Fisher Exact test for over-representation of the subset of 299 genes annotated as “Activation” within the “Components of CAR-T” sheet in the NanoString CAR T characterization panel (*LBL-10664-01_CAR-T_Characterization_Gene_List.xlsx*, NanoString Inc.).

Kinetic assessments of DARIC33 activity after rapamycin removal

DARIC33 T cells were cultured with media or media supplemented with 1 nM rapamycin for 24 hours, washed three times in PBS and then cultured in fresh media for various times prior to removal of aliquots for analysis. Residual DARIC33 activity was assessed by cytokine production following stimulation with MV4-11 tumor cells (2:1 effector:target ratio) for 24

hours. The decrease in cytokine production was fit to a model of one phase exponential decay using GraphPad (Prism).

Clinically Appropriate DARIC33 Cell Manufacture

Closed system manufacturing of T cell products at clinical scale and using good medical practice-appropriate methods and materials was conducted essentially as described[7], with the following modifications. In a 100cm^2 gas-permeable GREX culture device (G-Rex100M-CS, WilsonWolf) containing X-Vivo15 media (Lonza) supplemented with 2% v/v KnockoutSR (Gibco) and 5ng/L rhIL7, 0.5ng/L rhIL15 and 1ng/L rhIL21 (Miltenyi), 65 x 10^6 CD4 and 65 x 10^6 CD8 T cells were stimulated with GMP Dynabeads CD3/CD28 CTS (Thermo Fisher) and transduced 24 hours later via spinoculation in a SEPAX C-Pro device (Cytiva) by addition of 0.1mg/mL protamine sulfate (APP Pharmaceutical) and concentrated lentiviral vector at an MOI or 2-3. Dynabeads were removed on day 7 of culture using a Dynamag CTS magnet. Cultures were propagated for a further 3-4 days until harvest. After a total culture time 10 or 11 days cells were concentrated by centrifugation, and resuspended in Crostor-CS5 for cryopreservation.

Assessment of DARIC33 activity in whole blood

T cells were co-cultured with MV4-11 tumor cells at a 1:1 effector to target ratio and then immediately centrifuged and resuspended in serial dilutions of rapamycin prepare in heparinized whole blood from healthy human volunteers (BloodworksNW) or from NSG mice (BIOIVT). After 24 hours, plasma was isolated and cytokine production was assessed using MesoScale Discovery cytokine assays. Cytokine production was normalized to the maximum observed in each donor, and dose-response curves fit using GraphPad.

Measurement of rapamycin concentrations in mouse blood

Mouse whole blood samples were treated with K_2EDTA to prevent clotting and stored at -80°C. Samples were analyzed for the concentration of rapamycin using a mouse whole blood qualified LC-MS/MS method performed by Charles River Laboratories, Inc. (Shrewsbury Site, 334 South Street, Shrewsbury, MA 01545).

Assessment of red blood cell partitioning of rapamycin in mouse blood

A red blood cell partitioning assay was performed by Charles River Laboratories, Inc (One Innovation Drive, Biotech 3 Worcester, MA 01605) and samples analyzed for the concentration of rapamycin using the qualified LC-MS/MS method (as described above).

Fresh (unfrozen) NSG mouse whole blood (treated with K_2EDTA) and plasma prepared from the blood were used in the assay. The hematocrit of the whole blood was determined. The matrices (whole blood and plasma) were warmed to 37°C for at least 20 minutes prior to use in the assay. The acetonitrile-diluted stock solutions were spiked (1.2 µL) into all matrices (598.8 µL) to final assay concentrations of 2 µM for rapamycin and methazolamide, each in triplicates. Immediately after spiking, an aliquot of rapamycin (100 µL) was removed, and flash frozen in a -80°C freezer. An aliquot of the methazolamide control (30 µL) was removed and quenched with 180 µL of ice-cold acetonitrile containing internal standards to precipitate proteins and then stored in a -20°C freezer (these samples were designated as T0 and serve as references for stability).

The assay plates containing the spiked matrix were incubated at 37°C for 1 hour with shaking. After the incubation period, the whole blood assay plates were centrifuged (15°C minimum) at 3500 rpm (~2643 x g) for 15 minutes (with no brake). Rapamycin aliquots (100 µL) were removed from the assay samples (i.e., the spiked plasma, and the plasma prepared by

centrifuging the spiked whole blood) and flash frozen in a -80°C freezer. Methazolamide aliquots (30 µL) were removed from the assay samples (i.e., the spiked plasma, and the plasma prepared by centrifuging the spiked whole blood) and quenched with 180 µL of ice-cold acetonitrile containing internal standards.

Rapamycin samples were stored frozen at -80°C until analysis. Quenched samples were vortex-mixed, and then centrifuged at 3100 rpm (2074 x g) for 10 minutes at approximately 4°C. Whole blood stability samples were sonicated for 15 minutes prior to centrifugation. Supernatant (50 µL) was removed and transferred to new 96-well microtiter plates and diluted with 100 µL of water. Samples were stored refrigerated until analysis.

Mean peak area ratios were used to calculate the partitioning ratio and coefficient according to the following equations:

Concentration Ratio (Adjusted for Hematocrit) = Mean peak area of spiked plasma / Mean peak area of plasma from spiked whole blood x 1/(1-H), where H=% hematocrit.

Partitioning Coefficient (Kp(RBC/Plasma)) = [(Mean peak area ratio of spiked plasma / Mean peak area of plasma from spiked whole blood) -1] x (1/H) +1

Bound (%) = 1-[((Mean peak area of plasma from spiked whole blood x (1-H)) / Mean peak area of plasma)] x 100%

Plasma/Blood ratio = (Concentration in spiked plasma) / (Concentration in plasma from spiked whole blood)

Adjusted Plasma/Blood ratio = Plasma/Blood ratio x [1/(1-H)], where H is % hematocrit

Partitioning Coefficient (Kp) = (Plasma/Blood ratio -1) x 1/H+1

RBC Partitioning or Binding is indicated if the Adjusted Plasma/Blood ratio is >1, and if Kp is >0.

Stability (%) was calculated as mean peak area at T1hour / Mean peak area at T0 x 100%.

Assessment of plasma protein binding of rapamycin in mouse and human serum

Plasma protein binding of rapamycin was evaluated using an ultracentrifugation method performed by Charles River Laboratories, Inc (One Innovation Drive, Biotech 3 Worcester, MA 01605) and samples analyzed for the concentration of rapamycin using the qualified LC-MS/MS method (as described above).

Individual stock solutions of rapamycin (test article) and warfarin (control article) were prepared at 10 mM in DMSO and were further diluted to 1 mM with DMSO.

Frozen matrices (CD-1 mouse, NSG mouse and human plasma (K2EDTA)) were thawed and then centrifuged at 3100 rpm for 10 minutes at 4°C to remove particulates. Each matrix was warmed (37°C) for at least 10 minutes and then the pH of each matrix was checked and adjusted to 7.4 with 10% phosphoric acid or 1N sodium hydroxide, as necessary.

The 1 mM stock solutions were spiked into each matrix to a final assay concentration of 2 μM for rapamycin and warfarin. The final DMSO concentration in each matrix was 0.2%.

To measure rapamycin stability in plasma, compound-spiked matrices were incubated at 37°C for 10 minutes (to provide time to reach the estimated binding equilibrium). After incubation, an aliquot (rapamycin:100 μL, controls:15 μL) of the compound-spiked matrix was removed from each matrix and were transferred to a 96-well plate, matrix matched with an equal volume of 1X PBS. The control compounds were then quenched by the addition of 180 μL of ice-cold acetonitrile containing internal standards (carbutamide, glyburide and chrysin at 250

ng/mL). These samples are designated as T0 and serve as references for stability in matrix. Quenched samples were sealed and stored refrigerated until ultracentrifugation was completed.

To assess the extent of rapamycin protein binding, after incubation to reach binding equilibrium, 0.5 mL of spiked matrix was transferred into polycarbonate ultracentrifuge tubes, and then placed into a Beckman TLA-100.4 rotor pre-warmed to 37°C. Samples were centrifuged at approximately 100,000 rpm for 2.5 hours at 37°C with lowest brake setting. Concurrently, the remaining compound-spiked matrices were incubated at 37°C ("Total" analyte sample).

Following the ultracentrifugation, the supernatant (rapamycin:100 µL, control:15 µL) was transferred to a microtiter plate containing an equal volume of blank matrix. These samples are referred to as "Supt free" in the results. From the concurrently incubated (non-centrifuged) spiked matrices, an aliquot (rapamycin:100 µL, control:15 µL) was removed and matrix-matched with an equal volume of PBS. All matrix-matched control samples were quenched with 180 µL of ice-cold acetonitrile containing internal standards (carbutamide, glyburide and chrysin at 250 ng/mL).

Matrix-matched rapamycin samples (100 µL) were flash frozen at -80°C until analysis.

Quenched control samples were centrifuged at 3100 rpm for 10 minutes at 4°C to sediment the precipitated protein. An aliquot of the supernatant (50 µL) was transferred to a new microtiter plate and diluted with water (100 µL). Samples were stored refrigerated until analysis.

Data were captured and processed using Analyst v.1.6.2 (AB Sciex). Data were analyzed and results were calculated using Microsoft Excel.

Calculations:

$$\% \text{ Free} = \frac{\text{Peak Area Ratio of Supernatant}}{\text{Peak Area Ratio of Total}} \times 100$$

The ± value for calculated % Free =

$$\text{Mean \% Free} \times \sqrt{\left(\frac{\text{SD of Mean Peak Area Ratio of Supernatant}}{\text{Mean Peak Area Ratio of Supernatant}}\right)^2 + \left(\frac{\text{SD of Mean Peak Area Ratio of Total}}{\text{Mean Peak Area Ratio of Total}}\right)^2}$$

$$F_u \text{ (Unbound fraction)} = \frac{\text{Peak Area Ratio of Supernatant}}{\text{Peak Area Ratio of Total}}$$

$$\% \text{ Bound} = 100\% - \% \text{ Free}$$

$$\% \text{ Stability} = \frac{\text{Mean Peak Area Ratio after Incubation}}{\text{Mean Peak Area Ratio } T_0} \times 100$$

Pediatric rapamycin exposure modeling

A 2–compartment pharmacokinetic population PK model of sirolimus for pediatric patients receiving blood and marrow transplantation provided a model and estimated parameters[8]. Simulations were done in Nonmem and graphed in R. Estimates of inter-subject variability parameters were derived from a second published model of sirolimus in cancer patients[9]. The model used a calculated clearance (Cl) dependent on BSA **(**CL = 6.6 *(BSA/1.14), incorporating Cl of 6.6 L/h[8]. BSA dependency of rapamycin PK parameters has previously been reported[10]. Average age of pediatric patients reported in Goyal et al[8] is 10.1 years old but BSA levels were not reported. The average BSA levels for this age group is 1.14 sq.m. (https://www.calculator.net/body-surface-area-calculator.html).

Flow cytometry

Immunophenotyping of DARIC33 T cells was performed using standard staining and flow cytometry techniques. Cells were stained with combinations of the following fluorophore-conjugated anti-human monoclonal antibodies and live/dead viability dye (Invitrogen, catalog L23105) according to manufacturer's instructions. Data was acquired on a LSRFortessa (BD Biosciences) and flow cytometric analysis was performed using FlowJo (FlowJo, LLC).

Surface expression of DARIC33 components was quantified after 24-hour incubation with 1nM rapamycin by staining using a soluble CD33 antigen (ACRO, catalog CD3-H82E7), conjugated to Streptavidin-APC (Biolegend, catalog 405207), MonoRab™ Rabbit Anti-Camelid VHH (Genscript, catalog A01994-200), and Anti-FRB (custom reagent, Olympic Protein Technologies). Surface expression of CD19 CAR was quantified using biotinylated CD19 CAR detection reagent (Miltenyi Biotec, catalog 130-115-965) and Streptavidin-APC (Biolegend, catalog 405207). Additional characterization of T cell products used combinations of the following fluorochrome antibodies available from Biolegend: CD8a (clone RPA-T8, catalog 301040), CD4 (clone L200, catalog 562658), CD45RO (clone UCHL1, catalog 564291), and CD62L (clone DREG-56, catalog 304806).

Fluorescence resonance energy transfer (FRET) analysis of DARIC33 dimerization

For flow cytometry-based FRET analysis of DARIC33 dimerization, the T cells were stained with a PE-labeled anti-VHH antibody and an AlexFluor647-labeled anti-FRB antibody. The cells were treated with different concentrations of rapamycin and the FRET signal was detected in the Pe-Cy5 channel within the gated VHH/FRB dual-positive cells. Cell signal was acquired using the BD Fortressa and analyzed with FlowJo software. Rapamycin dosing and time post-rapa administration is described in the figure legend.

Quantification of CD33 antigen density

Surface CD33 antigen density was determined using flow cytometry. AML and control cell lines were labeled to saturation with anti-CD33-PE (Clone WM53, Biolegend) and the resulting geometric mean intensity of each sample was fit to a standard curve generated from the acquisition of a set of four beads labeled with known quantities of PE molecules (BD Quantibrite kit) using the same cytometer settings and following the manufacturer's instructions. The resulting number of PE molecules per cell was then converted to antigen binding capacity using the fluorescence/protein ratio for the respective antibody lot provided by the supplier.

Analysis of CD33m expression in AML transcriptomes and healthy tissues

RNA-Seq FASTQ sequences generated from blood or bone marrow samples were downloaded from the NCBI Short Read Archive for the following AML cohorts: Lavallee et al., 2016[11]; Papaioannou et al., 2019 [12] ; Lux et al., 2021 [13]; and Abbas et al., 2021[14]. Sequences were aligned to the hg38 human genome reference[2] using STAR[3] and gene and transcript expression was quantified in TPM using RSEM[4]. Samples with ≥ 2 million uniquely aligned reads and ≥ 70% uniquely mapped reads were retained for subsequent analysis. In addition, Ensembl transcript-level expression (TPM) of CD33 isoforms were obtained for the TCGA AML (Cancer Genome Atlas Network et al., 2013; PMID: 23634996) and GTEx healthy tissue[15] cohorts from the UCSC Xena Toil Recompute TCGA+TARGET+GTEX dataset (*RSEM tpm UCSC Toil RNA-seq Recompute*) dataset[16].

Splice junction-based estimates of the proportion of CD33 transcripts comprised of CD33m (which lacks Exon 2) for the Lavallee et al. (2016) and Papaioannou et al (2019) cohorts were computed using junction counts from STAR *SJout.tab* files generated during the alignment. The relevant splice junctions are (all on the forward strand): "SkipExon2" = chr19:51225156 -

51225802; “Exons2-3” = chr19:51225599 – 51225802; and “Exons1-2” = chr19:51225156 – 51225217. The percentage of CD33 that is CD33m (lacks Exon 2) is estimated as:

100 X [Counts SkipExon2] / ([Counts SkipExon2] + 0.5 X ([Counts Exons1-2] + [Counts Exons2-3))

(Where the “0.5” in the denominator of the above equation prevents double-counting of the CD33M transcript).

Ensembl transcript-based estimates of CD33m (lacking Exon2) and CD33M (containing Exon 2) expression levels were computed for all AML cohorts were computed from Ensembl Transcript TPM levels as follows:

CD33m (Lacking Exon 2) [TPM] = ENST00000421133 [TPM] + ENST00000436584 [TPM]

CD33M (Containing Exon 2) [TPM] = ENST00000262262 [TPM] + ENST00000391796 [TPM]

Similarly, the Ensembl transcript-based estimates of the proportion of CD33 transcripts comprised of CD33m was computed:

100 X CD33m (Lacking Exon 2) [TPM]/(CD33m (Lacking Exon 2) [TPM] + CD33M (Containing Exon 2) [TPM])

SNP genotyping analyses of sorted genomic bam files from alignments of the Lavallee et al. (2016) and Papaioannou et al (2019) datasets for rs12459419 C>T and rs2455069 A>G were performed using *samtools mpileup*.

To relate RNA-Seq-based predictions of CD33 SNP genotypes to CD33m splicing frequencies, samples were filtered to include only those that had at least two counts at the allele of interest (from *samtools mpileup*) and at least 5 splice junction counts proximal to Exon 2 (“SkipExon2”+”Exon1-2”+ “Exon2-3” ≥ 5 TPM) or at least 5 TPM total for CD33 (CD33m [TPM] + CD33M [TPM]) for junction-based or Ensembl transcript-based correlations respectively. For visualization, samples with reference allele frequencies of exactly 0 or exactly 1 were binned “HOM Alt (est)” or “HOM Ref (est)” respectively, and samples with reference

allele frequencies > 0 and < 1 were binned "HET (est)", where "est" indicates that these are genotype predictions based on SNP genotyping from RNA-Seq. Spearman correlation statistics were computed directly between the SNP genotyping reference allele frequency estimates (not the binned values used for visualization).

Rapamycin population pharmacokinetic modeling

The analysis was performed using a non-linear mixed effects modeling approach with a qualified installation of Phoenix NLME version 8.3.5[17]. The statistical computing program R (www.r-project.org, version 4.2.2) was used in the pre- and post-processing of data and model outputs. The package ggplot2 was used to generate visual representations.

A previously published population pharmacokinetic (PopPK) model describing the pharmacokinetics of rapamycin in pediatric blood and marrow transplantations[8] was adapted for use within the Phoenix NLME software. The model was a two-compartment model with oral absorption and first order clearance. Structural parameters included central volume (V), clearance (Cl), peripheral volume (V2), intercompartmental clearance (Cl2), and the absorption rate constant (Ka). Because no random effects were given for the PopPK model described above, we assumed that variability would be similar for adult and pediatric patients, and therefore incorporated random effects described from a previously reported model of rapamycin pharmacokinetics in adult patients[9]. Final model estimates for structural parameters of the pediatric model and random effects of the adult model were used to create simulations of pediatric rapamycin concentrations.

The published pediatric rapamycin PopPK model was adapted into Phoenix NLME and used for simulations of various doses and dosing regimens:

- Various simulated doses from 0.5 to 4 mg/kg.
- Various simulated dosing schema such as daily dosing, twice daily dosing, loading/maintenance dosing, etc.

Evaluation of patient blood, serum and chloroma samples

PLAT08 is an on-going phase 1 study of $CD4^+$ and $CD8^+$ T cells lentivirally transduced to express the DARIC33 transgene, delivered via intravenous infusion following lymphodepleting chemotherapy in pediatric and young adult patients (<30 years old) with relapsed or refractory acute myeloid leukemia. Following enrollment, $CD4^+$ and $CD8^+$ T cells isolated from cells collected by leukapheresis were combined in a 1:1 ratio to manufacture SC-DARIC33 as described[18].

Processing of patient biofluids

Patient serum was isolated from venous blood in additive-free collection tubes. Following incubation at room temperature for an hour, collection tubes were centrifuged at 1,000 g for 15 minutes before serum was collected, aliquoted and cryopreserved at -80°C.

Evaluation of patient serum using electrochemiluminescence assays

Healthy control plasma and patient serum samples were thawed and assessed for selected cytokines using V-PLEX Plus Cytokine Panel 1 Human, V-PLEX Plus Cytokine Panel 2 Human, and V-PLEX Plus Proinflammatory Panel 1 Human according to manufacturer instructions (Meso Scale Diagnostics, Cat. No. K15047G, K15084G, and K15049G respectively). All serum samples were diluted according to manufacturer recommendations. Following wash, standards and samples were added in duplicates to wells and incubated at room temperature for 2 hours with shaking. After wash, a proprietary SULFO-TAG conjugated detection antibody was added

to the wells and incubated at room temperature for 2 hours with shaking. After a final wash, the plate was developed using read buffer and analyzed immediately. Data was collected using MESO QuickPlex SQ 120 instrument using MSD Discovery Workbench version 4.0.13 analysis software. Standard curves were generated using 4-parameter logistic model. For the purposes of analyses, any value that was below the lowest limit of detection (LLOD) was considered undetectable.

To determine impacts of rapamycin exposure on T cell immunophenotypes, cryopreserved healthy donor or patient infusion products were thawed and rested overnight at 37°C in a humidified incubator in RPMI 1640 (Gibco) + 10% FBS (ATLAS) + 1% L-glutamine (Gibco) with or without 1nM rapamycin (SelleckChem) prior to evaluation by flow cytometry. Peripheral blood (PB) was evaluated fresh following red blood cell lysis (eBioscience). Single-cell suspensions were generated from unfixed tissue by mechanical dissociation and filtration without the use of enzymes. Immunophenotyping of PB, tissue and infusion cell products used standard staining and flow cytometry techniques, and following fluorescent reagents: fixable viability stain (FVS510, BD #564406), CD3-BUV395 (BD #563546), CD27-BUV737 (BD #612829), PD-1-BV421 (BD #564323), CD8-BV605 (BD #563116), TIM3-BV785 (BioLegend #345032), CD101-PE (BD #566371), CD33-PE-Cy7 (BioLegend #303434), CD4-R718 (BD #567092), CD137-APC/Fire750 (BioLegend #309834), anti-VHH-iF488 (GenScript #A01862) and custom APC conjugated anti-FRB[19] (Olympic Protein Technologies).

Study Approval

The PLAT08 study (NCT050105152) is conducted in accordance with FDA and international conference on harmonization guidelines for good clinical practice, the declaration of Helsinki and applicable institutional review board guidelines (study protocol approved by Seattle

Children's Institutional Review Board). All patients or their guardians provided written informed consent. Written informed consent was received for the use of photographs and the record of informed consent has been retained at Seattle Children's.

Statistical Analysis

Statistical analyses were performed using Prism (GraphPad), R or Python software. Results with a $p \leq 0.05$ after correcting for multiple comparisons were evaluated as statistically significant. Comparisons of means among more than two groups used one- or two-way ANOVA or t-tests corrected for multiple testing by the method of Benjamini-Hochberg as indicated in corresponding figure legends. When global differences were identified, follow-up pairwise comparisons were made, correcting for repeat testing using the method of Benjamini-Hochberg. In comparing repeated measures of DARIC T cell designs derived from multiple donors, two-way ANOVA was utilized, including the donor as a factor and correcting for multiple comparisons. Comparisons among the duration of tumor-symptom free survival of mice utilized the log rank test, adjusted for multiple comparisons using the Bonferonni method[20], except when indicated in the figure legends. Tumor growth rates defined as the slope of a best-fit line on the graph of log[Flux] vs days, were compared among various treatments using t-tests corrected for multiple testing by the method of Benjamini-Hochberg[21].

ADDITIONAL REFERENCES

1. Conant, D. *et al.* Inference of CRISPR Edits from Sanger Trace Data. *The CRISPR Journal* **5**, 123–130 (2022).
2. Schneider, V. A. *et al.* Evaluation of GRCh38 and de novo haploid genome assemblies demonstrates the enduring quality of the reference assembly. *Genome Res.* **27**, 849–864 (2017).

3. Dobin, A. *et al.* STAR: ultrafast universal RNA-seq aligner. *Bioinformatics* **29**, 15–21 (2013).
4. Li, B. & Dewey, C. N. RSEM: accurate transcript quantification from RNA-Seq data with or without a reference genome. *BMC Bioinformatics* **12**, 323 (2011).
5. Bausch-Fluck, D. *et al.* The in silico human surfaceome. *Proceedings of the National Academy of Sciences* **115**, E10988–E10997 (2018).
6. Seabold, S. & Perktold, J. Statsmodels: Econometric and Statistical Modeling with Python. in 92–96 (Austin, Texas, 2010). doi:10.25080/Majora-92bf1922-011.
7. Gardner, R. A. *et al.* Intent to treat leukemia remission by CD19CAR T cells of defined formulation and dose in children and young adults. *Blood* blood-2017-02-769208 (2017) doi:10.1182/blood-2017-02-769208.
8. Goyal, R. K. *et al.* Sirolimus Pharmacokinetics in Early Postmyeloablative Pediatric Blood and Marrow Transplantation. *Biol Blood Marrow Transplant* **19**, 569–575 (2013).
9. Wu, K. *et al.* Nonlinear Population Pharmacokinetics of Sirolimus in Patients With Advanced Cancer. *CPT: Pharmacometrics & Systems Pharmacology* **1**, 17 (2012).
10. Scott, J. R. *et al.* Population Pharmacokinetics of Sirolimus in Pediatric Patients With Neurofibromatosis Type 1. *Therapeutic Drug Monitoring* **35**, 332–337 (2013).
11. Lavallée, V.-P. *et al.* RNA-sequencing analysis of core binding factor AML identifies recurrent ZBTB7A mutations and defines RUNX1-CBFA2T3 fusion signature. *Blood* **127**, 2498–2501 (2016).
12. Papaioannou, D. *et al.* The long non-coding RNA HOXB-AS3 regulates ribosomal RNA transcription in NPM1-mutated acute myeloid leukemia. *Nat Commun* **10**, 5351 (2019).

13. Lux, S. *et al.* Deregulated expression of circular RNAs in acute myeloid leukemia. *Blood Adv* **5**, 1490–1503 (2021).
14. Abbas, H. A. *et al.* Decoupling Lineage-Associated Genes in Acute Myeloid Leukemia Reveals Inflammatory and Metabolic Signatures Associated With Outcomes. *Front Oncol* **11**, 705627 (2021).
15. GTEx Consortium. The GTEx Consortium atlas of genetic regulatory effects across human tissues. *Science* **369**, 1318–1330 (2020).
16. Goldman, M. J. *et al.* Visualizing and interpreting cancer genomics data via the Xena platform. *Nat Biotechnol* **38**, 675–678 (2020).
17. Download Phoenix 8.4. *Certara* https://www.certara.com/download-phoenix-8-4/.
18. Ceppi, F. *et al.* Modified Manufacturing Process Modulates CD19CAR T-cell Engraftment Fitness and Leukemia-Free Survival in Pediatric and Young Adult Subjects. *Cancer Immunology Research* **10**, 856–870 (2022).
19. Leung, W.-H. *et al.* Sensitive and adaptable pharmacological control of CAR T cells through extracellular receptor dimerization. *JCI Insight* **4**, (2019).
20. Miller, R. G. *Simultaneous Statistical Inference*. (Springer New York, New York, NY, 1981). doi:10.1007/978-1-4613-8122-8.
21. Benjamini, Y. & Hochberg, Y. Controlling the False Discovery Rate: A Practical and Powerful Approach to Multiple Testing. *Journal of the Royal Statistical Society: Series B (Methodological)* **57**, 289–300 (1995).
22. Nair-Gupta, P. *et al.* A novel C2 domain binding CD33xCD3 bispecific antibody with potent T-cell redirection activity against acute myeloid leukemia. *Blood Adv* **4**, 906–919 (2020).

SUPPLEMENTARY FIGURES

Supplemental Figure 1: CD33-specific VHH exhibit a range of affinities towards CD33 protein and produce functional DARIC33 lentiviral vectors.

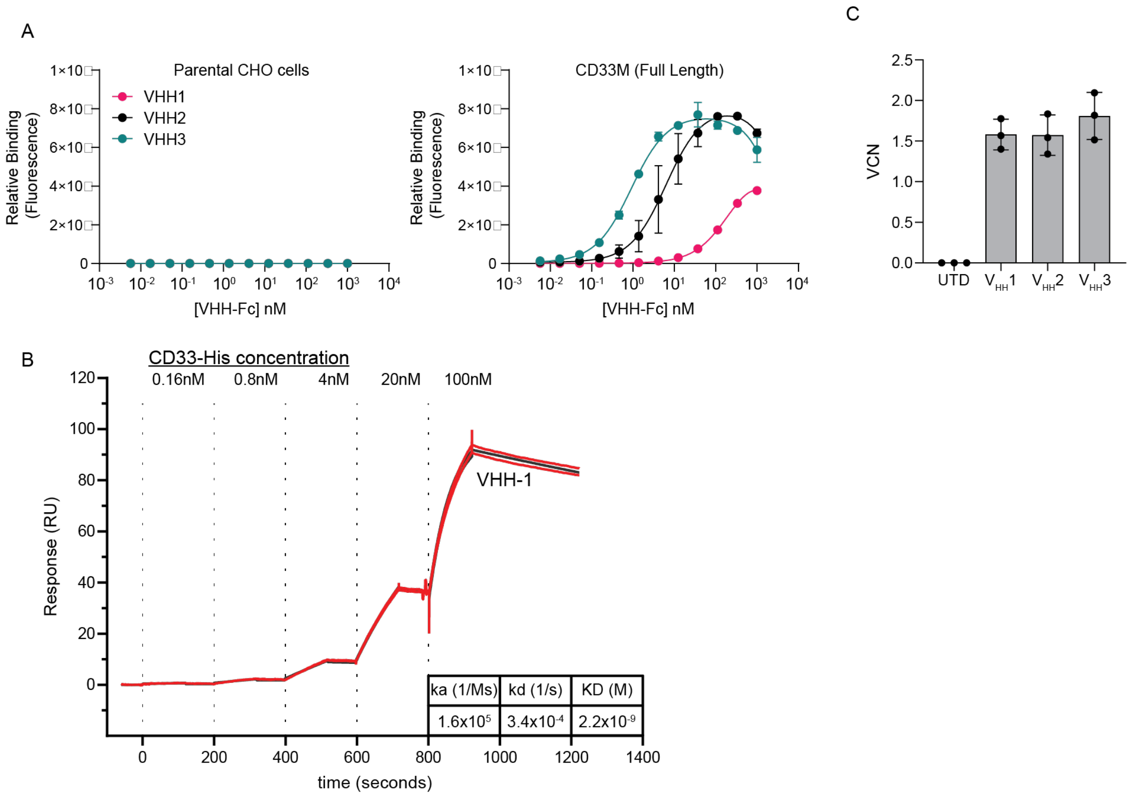

Figure S1. (A) Indicated concentrations of recombinant CD33-specific VHHs were incubated with parental CHO or CHO cells over-expressing full length CD33 isoform. Binding was detected using flow cytometric analysis and secondary antibodies recognizing an Fc tag appended to the VHH domain. Relative affinity was determined by fitting mean fluorescent intensity (MFI) values to a single-site binding isotherm (MFI=B_{max}*[VHH-Fc]/(K_d+[VHH-Fc]) + NS*[VHH-Fc] + Background), where Kd is the dissociation constant, B_{max} is the maximum fluorescence intensity, and NS is the nonspecific binding slope using Graphpad. (**B**) single cycle binding kinetics of CD33 to VHH1-captured surface performed at the indicated concentrations.

Red line represents the observed bindings kinetics while the black line is kinetic fit of the data. Calculated kinetic constants are shown in lower left. Results are representative of 3 different runs with duplicate CD33 binding analysis. (**C**) Vector copy number (VCN) analysis of the T cells described in Figure 1 demonstrating comparable integration of vector sequences into T cell products.

Supplemental Figure 2: DARIC33 is highly responsive to both antigen expression and rapamycin dosing even in the presence of soluble antigen

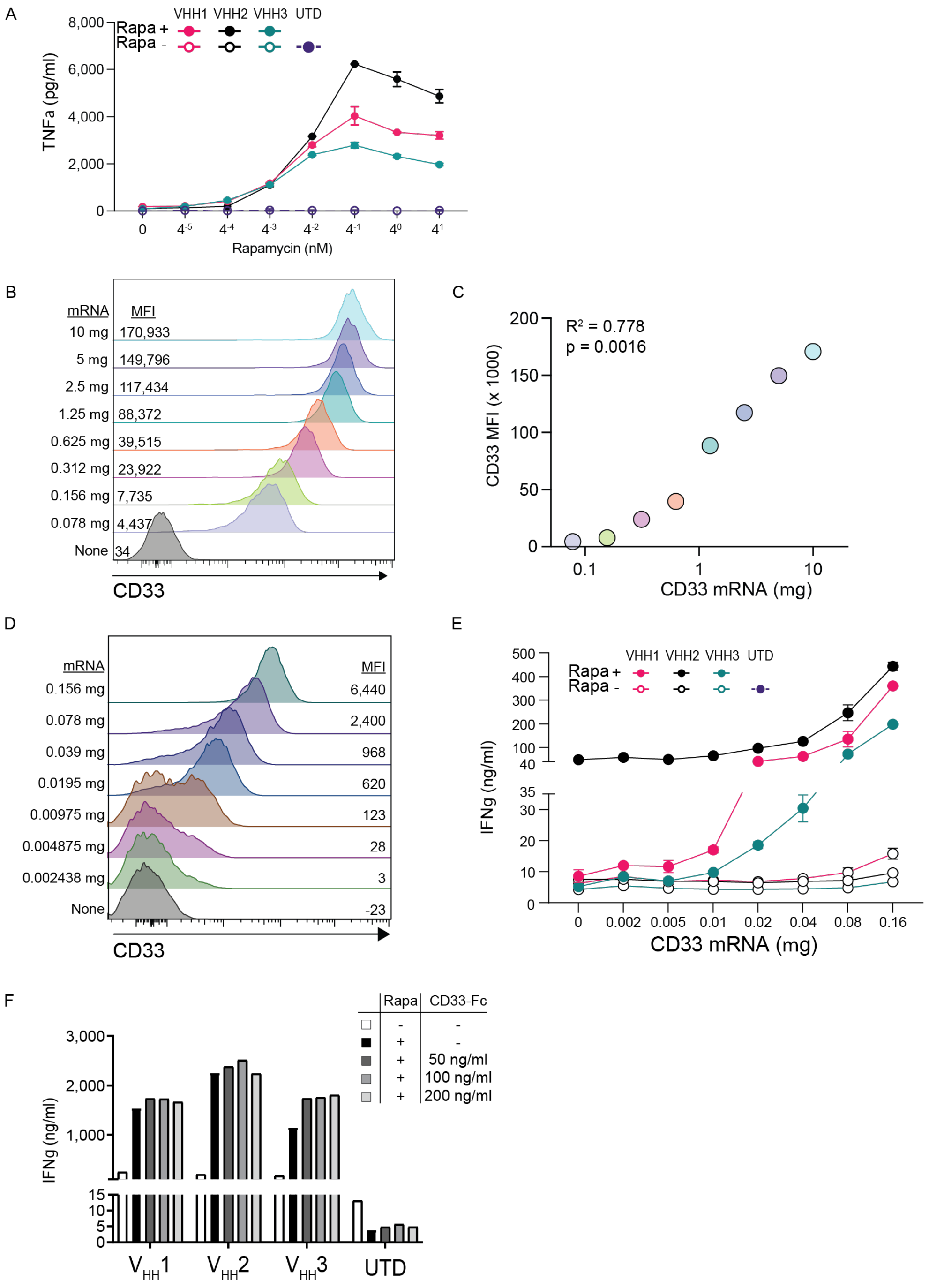

Figure S2. (A) T cells were cultured with MV4-11 in the presence of different concentrations of rapamycin as described in Figure 2 and TNFα production was analyzed by MSD. **(B)** 293T cells transfected with different amounts of CD33-encoding mRNA and CD33 expression analyzed by flow cytometry. **(C)** Correlation between mRNA concentration and CD33 MFI expression. Pearson's R^2 and corresponding p value. **(D)** 293T cells transfected with very low amounts of CD33 mRNA and analyzed by flow cytometry. **(E)** Cytokine following coculture of HEK293 T cells electroporated with very low amounts of CD33 mRNA (as in *D*) and UTD (control) or DARIC33 T cells containing different V_HH binders following coculture with or without rapamycin (rapa). **(F)** Cytokine release by UTD (control) or DARIC33 containing different V_HH binders following coculture with MV4-11 with or without rapamycin (rapa) plus increasing amount of recombinant CD33-Fc soluble antigen. The highest concentration (200ng/mL) is approximately a 100-fold excess above the amount of soluble CD33 present in healthy subjects and AML patients[22].

Supplemental Figure 3: DARIC33 T cells are specific for CD33 and can modulate growth of an antigen-low tumor model.

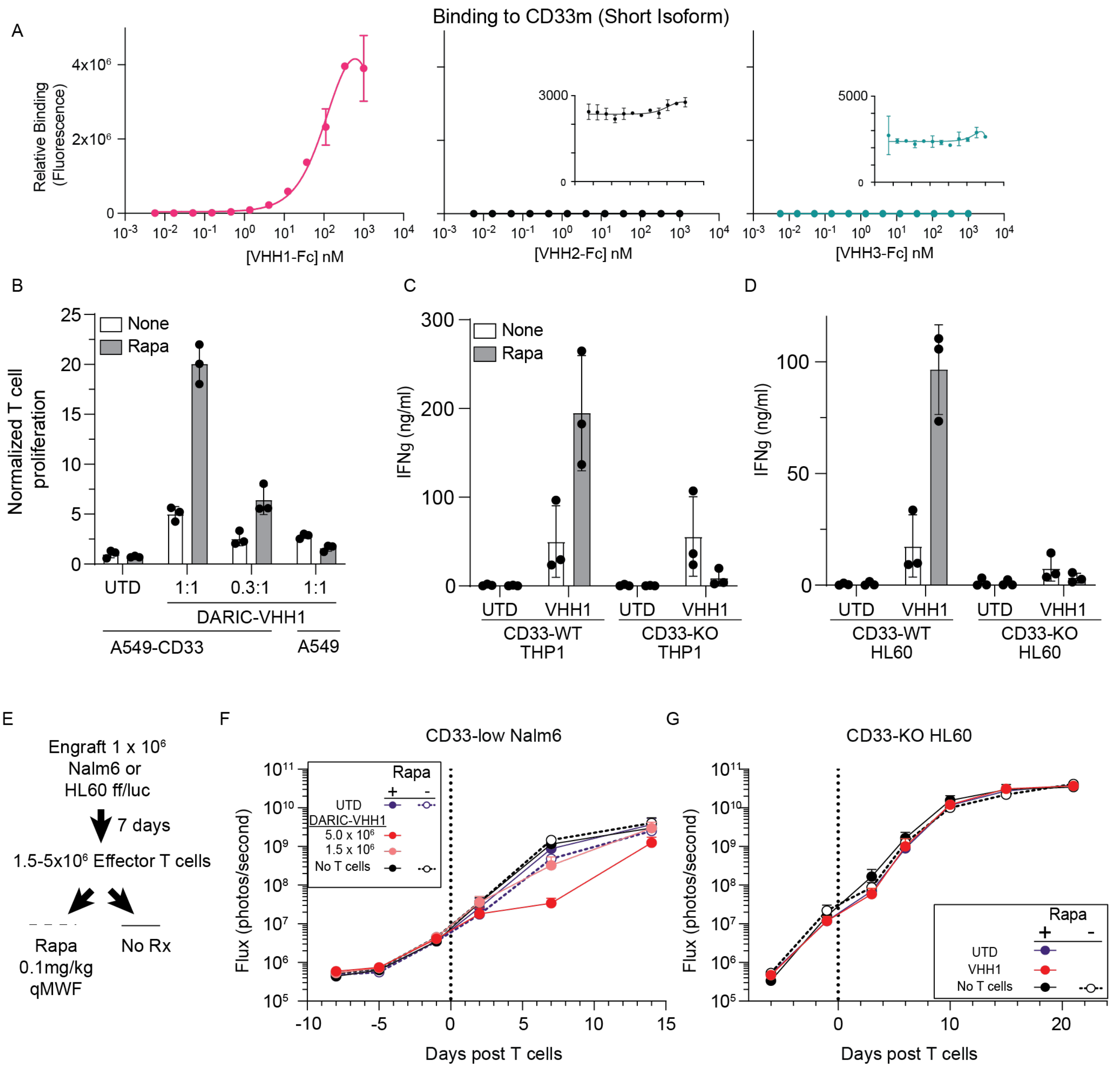

Figure S3. (A) Indicated concentrations of recombinant CD33-specific VHHs were incubated with parental CHO or CHO cells over-expressing the short CD33m isoform. Relative affinity was determined by flow cytometric binding analysis. Insert charts for VHH2 and VHH3 are zoomed-in analysis of binding to CD33m. (**B**) DARIC33 T cells were co-cultured with parental

or CD33+ A549 spheroids in the presence or absence of rapamycin and the number of T cells were counted by flow cytometry after 7 days of culture. DARIC33 T cells were co-cultured with WT or CD33-deficient (**C**) THP1 or (**D**) HL60 cell lines in the presence or absence of rapamycin and cytokine production was analyzed by MSD. (**E**) Schematic for *in vivo* analysis of DARIC33 activity against (**F**) CD33-low Nalm6 tumors or (**G**) CD33-deficient HL60.CD33KO tumors.

Supplemental Figure 4: A single V_HH binder is specific for the short CD33m isoform, which is broadly expressed in AML samples

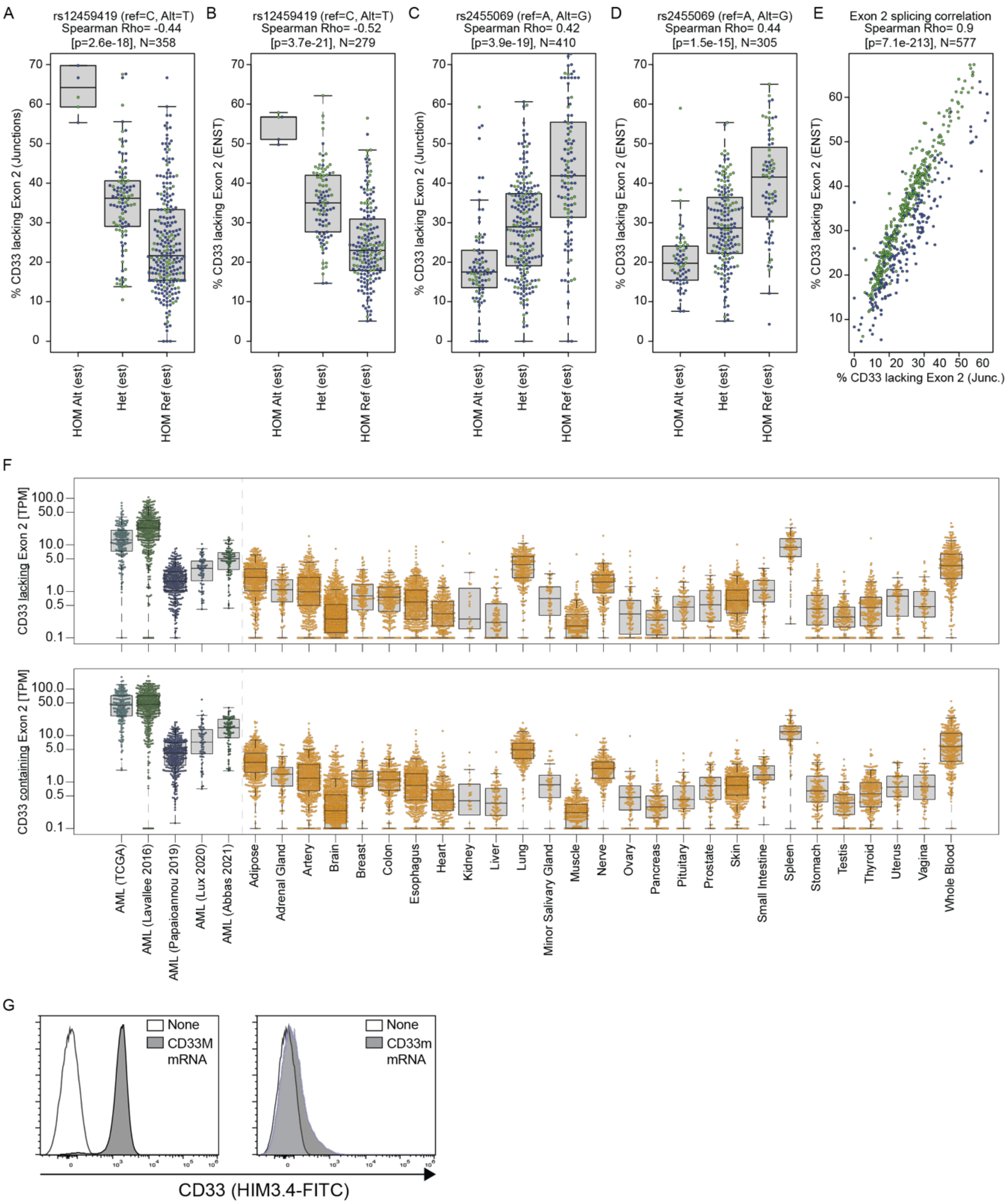

Figure S4. (A-F) Impact of CD33 SNPs on CD33 isoform expression in AML patient samples. The percentage of CD33 transcripts made up of the CD33m isoform (that lacks exon 2) was

estimated directly from bulk RNA-Seq splice junction counts (**A, C**) and from relative expression of Ensembl CD33 transcripts (**B, D**) for groups of patients predicted by RNA-Seq SNP genotyping to harbor different alleles of CD33 SNPs rs12459419 C>T (A-B) and rs2455069 A>G (**C-D**). Spearman rank correlation statistics between the proportion of CD33m splicing and the predicted reference allele frequency from SNP genotyping are shown. Colors indicate AML cohort (green = Lavallee et al. 2016, blue=Papaioannou et al. 2019). **(E)** Consistency between estimates of CD33m splicing prevalence based on splice junctions (x-axis) and Ensembl transcripts (y axis) in the two AML cohorts. Colors are as in *B-E*. Spearman's *rho* = 0.9, p = 7.1e-213, n = 577 AML cases. **(F)** Comparative expression analysis of CD33m (which lacks Exon 2, *top*) and CD33M (which contains Exon 2, *bottom*) isoform levels in AML cohorts (left, blue and green colors) and in healthy tissues (right, orange colors, data from the Genotype-Tissue Expression (GTEx) consortium[15]. Transcript abundances are quantified in transcripts per million transcripts sequenced (TPM). (**G**) Staining of HEK293 cells expressing CD33M (left) or CD33m (right) with the HIM3.4 antibody.

Supplemental Figure 5: DARIC33 T cells exhibit a transcriptional expression profile specific to the presence of both antigen and rapamycin.

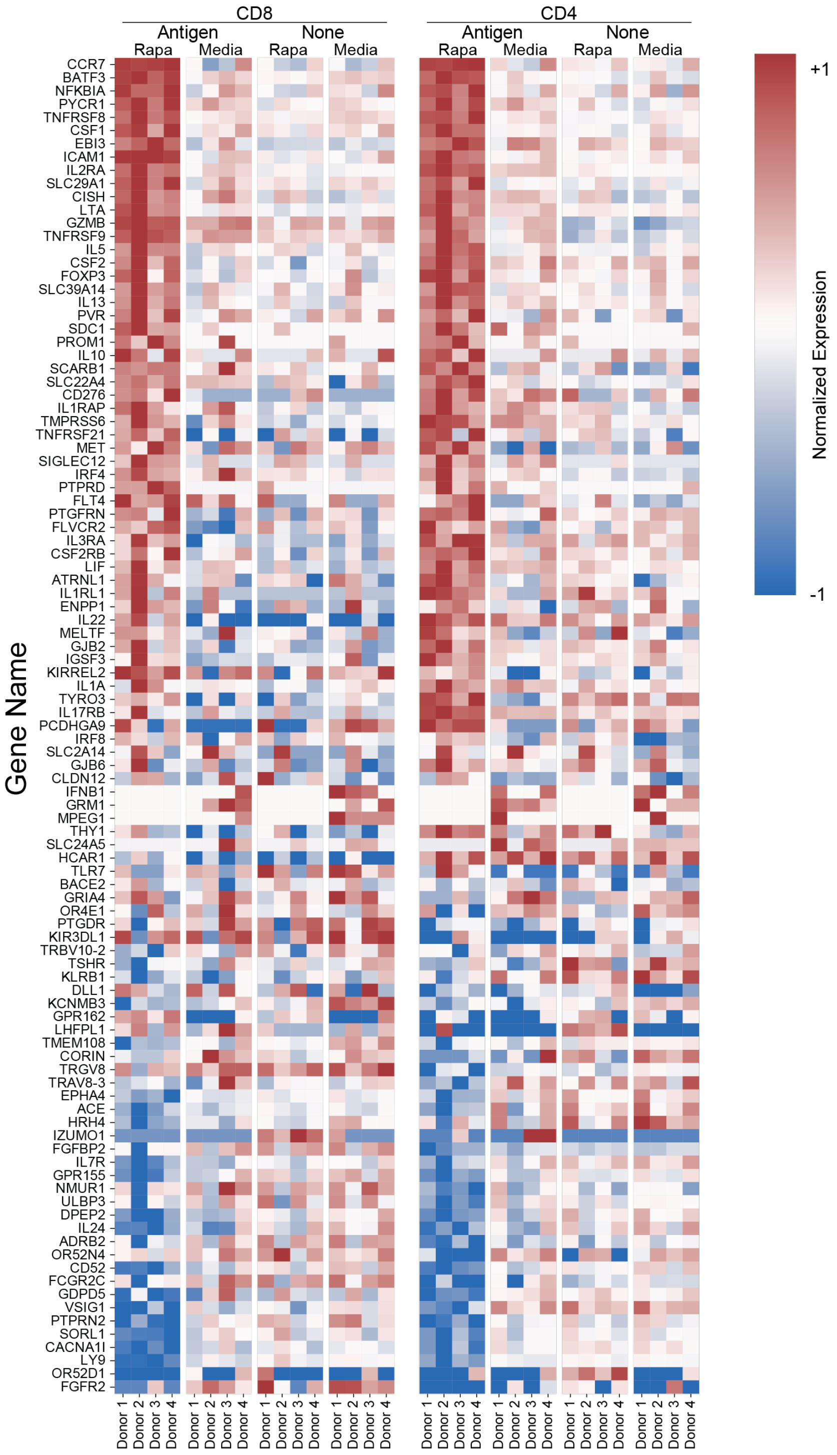

Figure S5. Heatmap of transcripts displaying significant ‘DARIC Active’ regulation among 4 conditions tested, (1) Antigen + rapamycin (i.e. *DARIC Active*), (2) Antigen alone, (3) rapamycin alone, or (4) media alone (control). Gene names are displayed on the vertical axis. CD8 T cell and CD4 T cell responses are shown on the left and right panels respectively. Colors indicates gene expression normalized to mean donor expression in the DARIC33 OFF condition (no RAPA, no Ag) scaled to max absolute expression for each cell population, where red indicates over-expression and blue indicates reduced expression compared to mean DARIC33 OFF. Genes are sorted by expression in the *DARIC33 Active* condition**.** The differentially expressed genes were selected based on their significance level (FDR corrected p-value <=0.05) and coefficient value (abs(coefficient) > 1.5)) in the linear-mixed model in at least one cell population as described in *Supplementary Methods*. *n = 4 donors.*

Supplemental Figure 6: DARIC33 T cells had similar functional activity and lower tonic signaling compared to CAR33 T cells

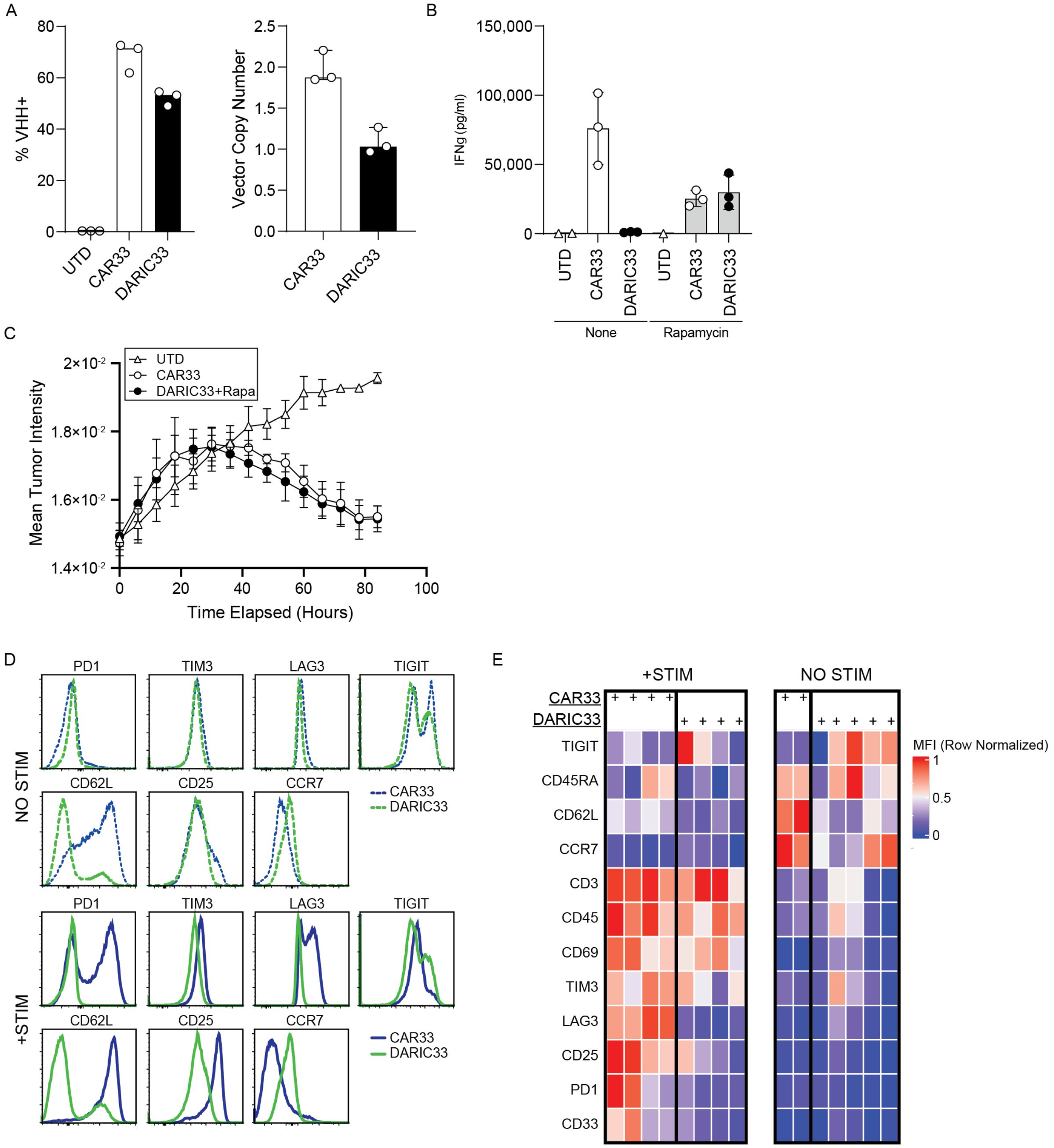

Figure S6. Donor matched CAR33 and DARIC33 T cells were generated and analyzed for (**A**) VHH expression and VCN. (**B**) CAR33 and DARIC33 T cells were co-cultured with CD33+

HL60 tumor cells and IFNg secretion analyzed by MSD. (**C**) Engineered CD33+ A549 cells were co-cultured with CAR33 or DARIC33 T cells and cytotoxicity evaluated through incucyte live cell imaging. (**D**) Phenotypic comparison of resting ("NO STIM) and stimulated ("+STIM") donor-matched CAR33 and DARIC33 cell products. Crypo-preserved donor-matched T cell products (n = 3) were thawed and incubated in either media alone ("NO STIM") or in media supplemented with 1 nM rapamycin followed by a 6 hour challenge with a three-fold excess of CD33+ K562 cells ("+STIM") prior to staining and analysis by flow cytometry. Histograms show expression levels of the indicated antigen after gating on VHH+ T cells. A representative donor is shown. (**E**) Heat map summarizing expression of the activation/exhaustion markers on CAR33 and DARIC33 cells at rest and following stimulation. To facilitate dynamic comparisons, color is scaled by row (e.g. row-normalized MFI).

Supplemental Figure 7: DARIC33 T cells drive cytotoxicity in a cell dose dependent manner.

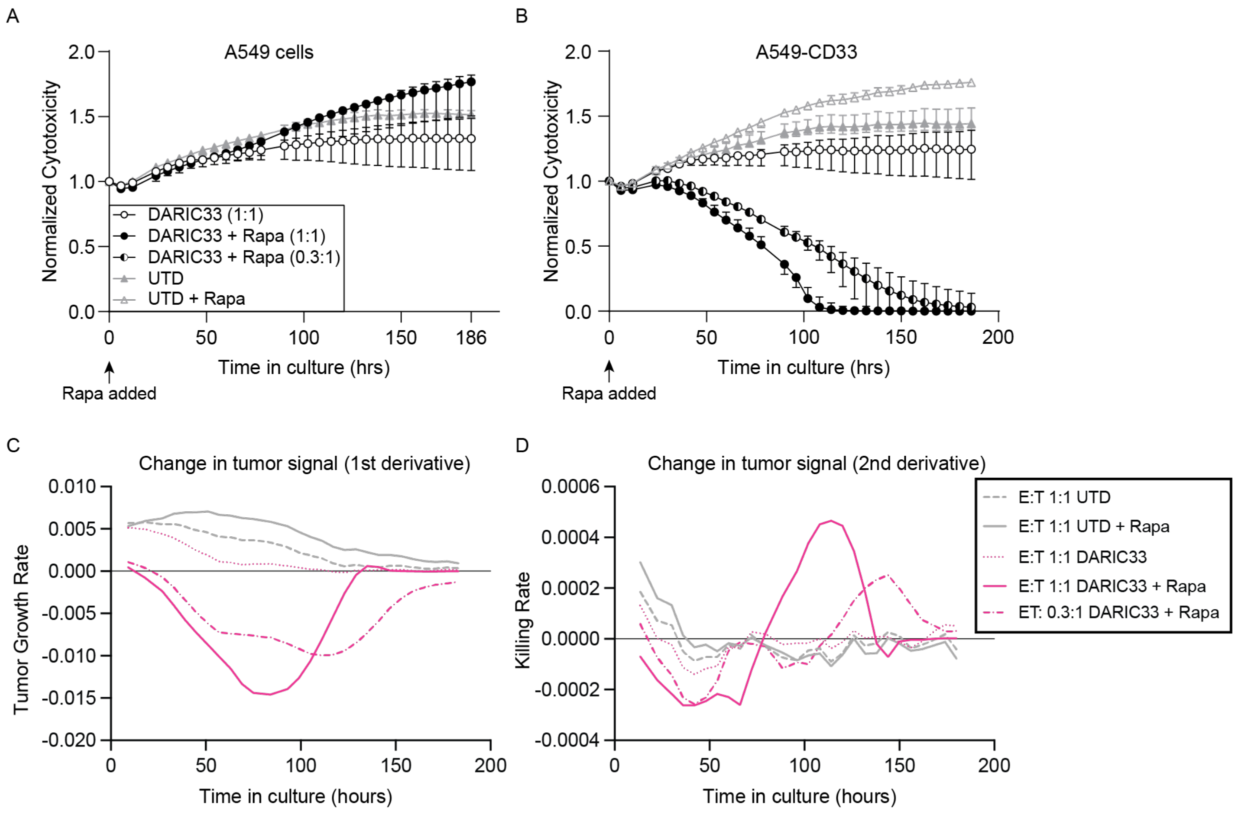

Figure S7. DARIC33 T cells were co-cultured at two different E:T ratios with parental **(A)** or CD33+ A549 cells **(B)** over-expressing NLR-mKate2 fluorescent protein. Cytotoxicity was analyzed by Incucyte live cell imaging system. **(C)** Spheroid target cell growth rate, graphed as the 1st derivative of the spheroid signal shown in *B*. **(D)** Spheroid target killing rate graphed as the 2nd derivative of spheroid signal shown in *B***)**. A sliding window of 5 adjacent points was used as a smoothing function. Killing is reflected in a negative second derivative, which occurs between the start of the graph and up to 75 hours in the case of DARIC33+Rapa with an effector to target ration (E:T) of 1:1. Positive growth rates shown in **(C)** among UTD, UTD + Rapa, and DARIC33 alone reflect absence of tumor killing and an increase in spheroid signal.

Supplemental Figure 8: FRET analysis demonstrates DARIC33 dimerization in the presence of rapamycin

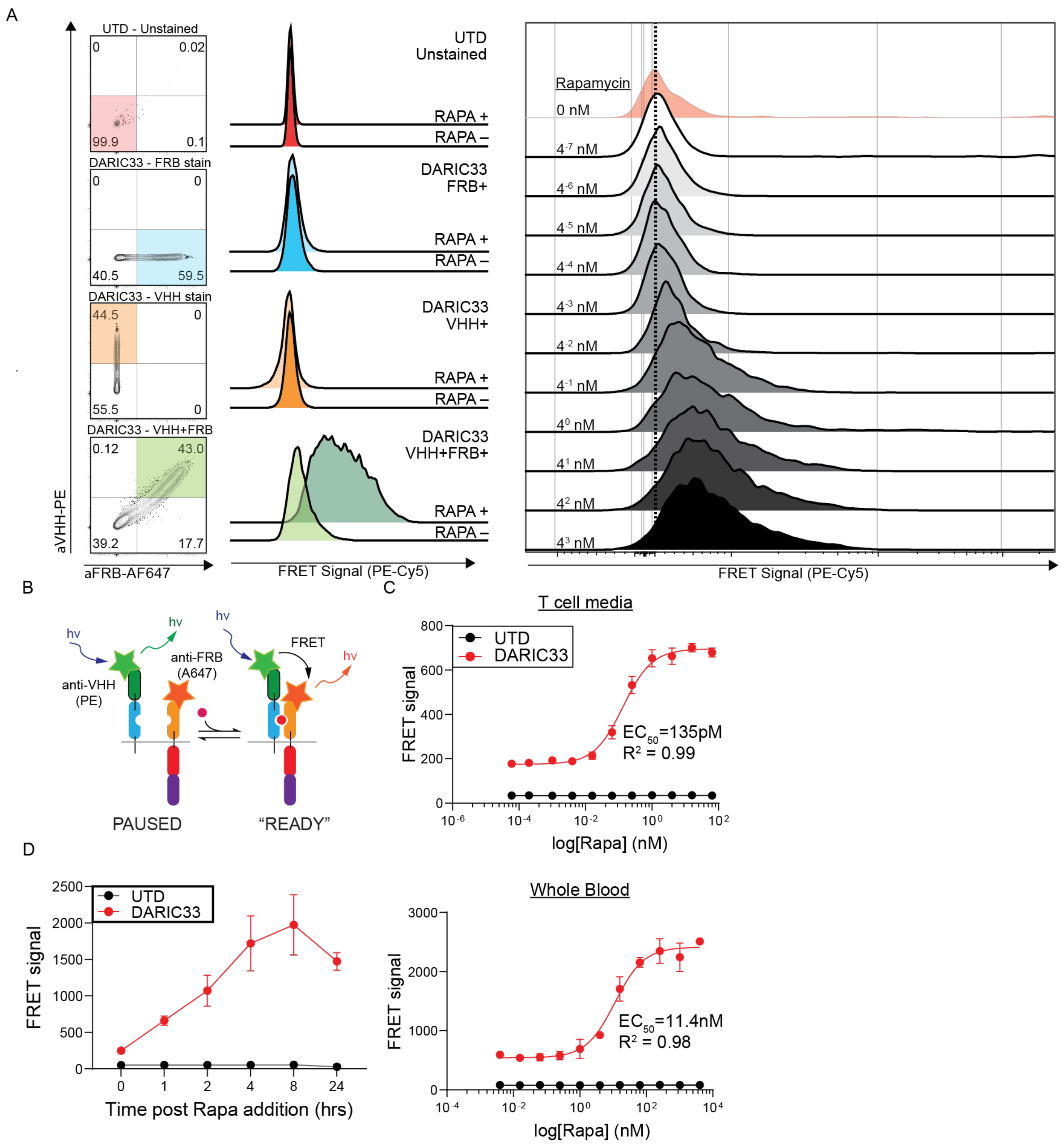

Figure S8. DARIC33 were stained with VHH and FRB-specific antibodies for FRET analysis. (**A**) Detection of the PeCy5 FRET signal solely within the dual FRB+/VHH+ subset. The FRET

signal was proportional to the rapamycin dose (right). (**B**) Schematic of FRET detection following dual antibody staining and rapamycin-mediated DARIC33 dimerization. **(C)** Concentration-dependent increase in the FRET signal following DARIC33 incubation with various doses of rapamycin when cultured in T cells media. **(D)** DARIC33 T cells were cultured in 1nM rapamycin and the FRET signal was analyzed at various time-points after rapamycin addition. (**E**) Concentration-dependent increase in the FRET signal following DARIC33 incubation with various doses of rapamycin when cultured in human whole blood.

Supplemental Figure 9: DARIC33 T cells control tumor growth in vivo over a wide range of rapamycin concentration

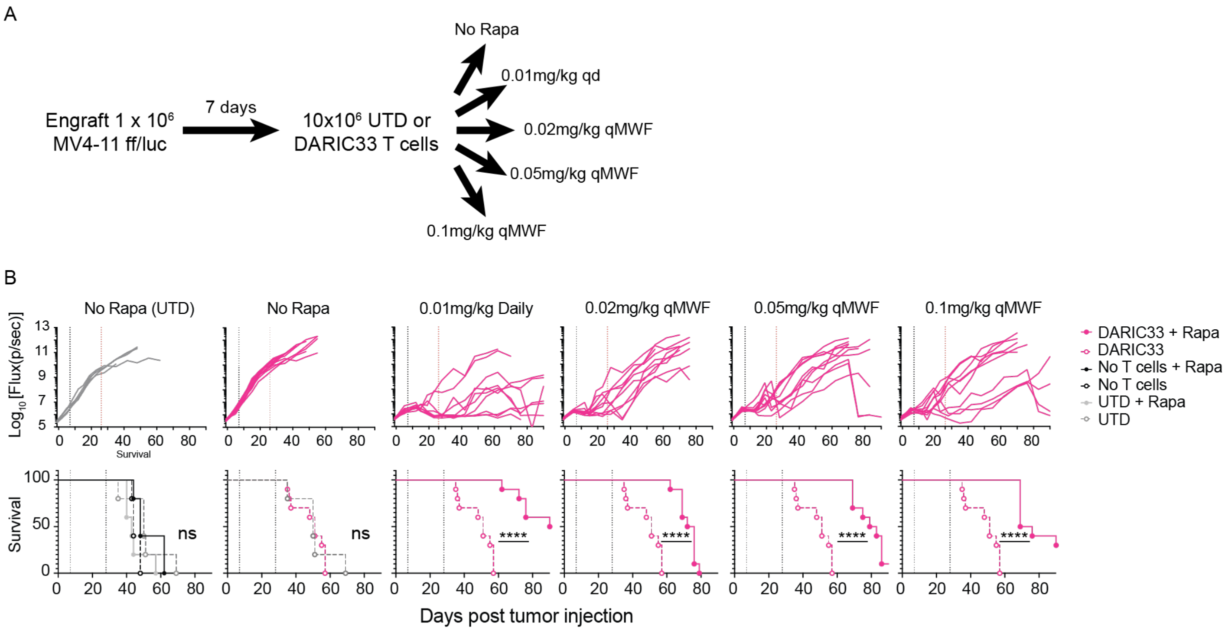

Figure S9. (A) Schematic of in vivo analysis of DARIC33 functionality with different rapamycin dose levels and administration schedules. **(B)** Tumor growth (top) and survival (bottom) of MV4-11 inoculated NSG mice treated with untransduced (UTD) or DARIC33 T cells followed by various rapamycin doses and schedules. Rapamycin was only continued for 21 days following tumor injection (last day of rapamycin administration is indicated by the right hand vertical dotted line). Tumor growth is shown as a spaghetti plot, one line for each mouse. Survival comparisons using thelog rank test are corrected for multiple comparisons by bonferroni's method. *n* = 5-10 mice per group. **** $p < 0.001$.

Supplemental Figure 10: Integration of preclinical models and simulated rapamycin dose exposure relationships reveals dosing strategy predicted to activate DARIC33 in vivo without reaching immunosuppressive concentrations.

A

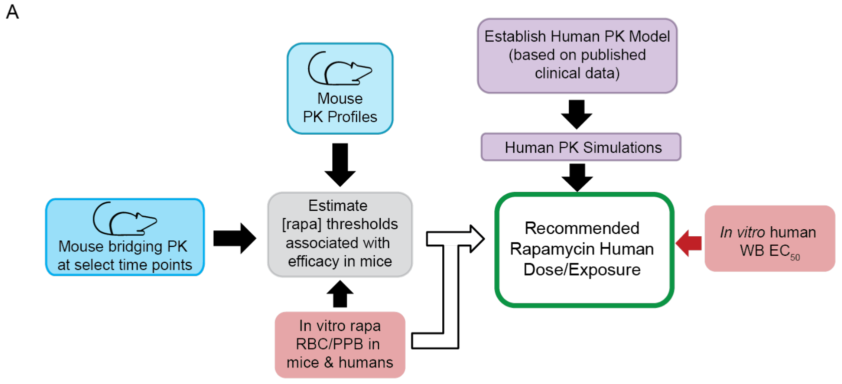

B

Parameter	Units	Value
Apparent Clearance (CL/F)	L/h	6.66
Volume of Distribution, central (V_C)	L/h	26.90
Volume of Distribution, peripheral (V_P)	L/h	630
Intercompartmental Clearance (Q)	L/h	4.62
Absorption rate constant (k_A)	h^{-1}	0.0535
IIV, CL		0.524
IIV, V_C		0.524
IIV, V_P		0.193
IIV, Q		0.705

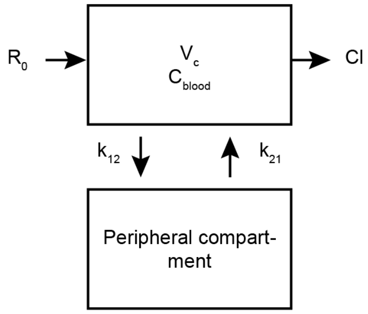

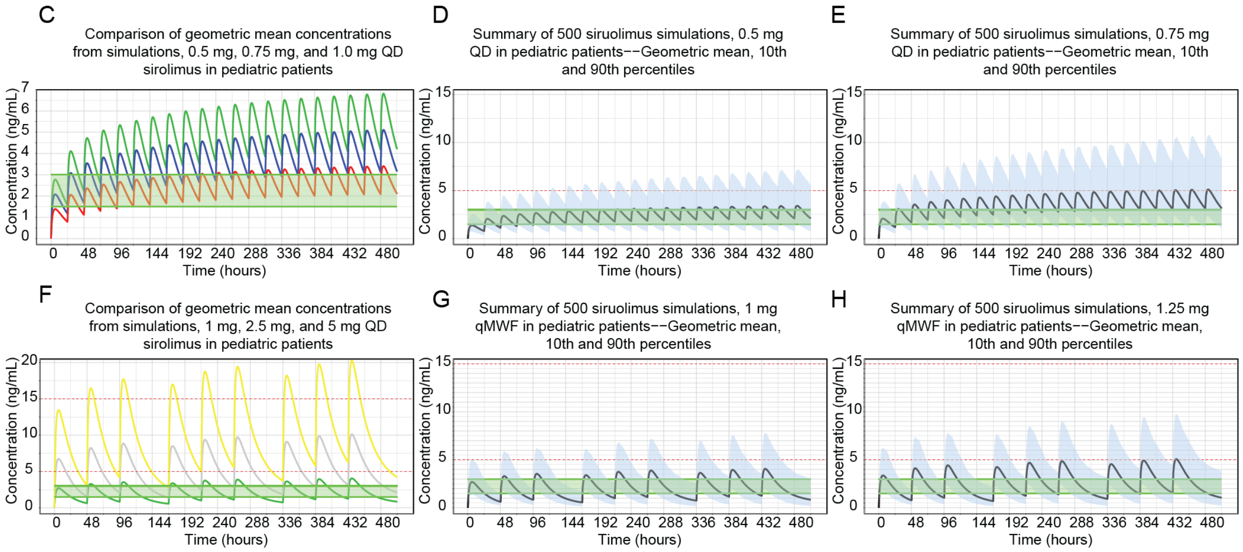

Figure S10. (**A**) Schematic describing strategy employed to determine recommended rapamycin dose based on exposure predicted in human patients and the range of targeted concentrations of rapamycin in blood. Human PK simulations are conducted as described in *Supplementary* Methods, drawing on population distributions of anthropomorphics and pharmacokinetics. (**B-D**) Graphs of blood concentrations of rapamycin over time following simulated rapamycin dose schedules. Once daily dosing (B – D) and three times weekly dosing (E-G) are shown. Panels B and E depict geometric mean blood concentrations for several doses, while panels C,D,F and G depict geometric mean and 10th, 90th percentiles of expected concentrations. Safe sirolimus trough concentrations for immunosuppression: 5-15 ng/mL (red dashed lines). The target sirolimus concentrations were defined as 1.5 – 3 ng/mL (shaded green).

ADDITIONAL SUPPLEMENTARY DATA ITEMS:

Supplemental Table 1: Quantification of endogenous and engineered CD33 expression on various cell lines. Cell lines in bold were used to test T cell functionality.

Supplemental Table 1

Cell Line	%CD33+	total ABC
A549 - CD33	100%	661,474
MOLM-13	100%	47,770
AML-193	100%	39,223
THP-1	100%	31,449
MOLM-14	100%	29,855
ML-1	100%	29,282
MV4-11	100%	28,879
Kasumi-3	100%	26,663
HL-60	100%	21,981
EOL-1	100%	21,163
U-937	100%	20,330
NOMO-1	100%	18,030
Kasumi-1	100%	9,009
OCI-AML3	99%	2,491
KG1a	39%	173
Nalm6	20%	141
BDCM	26%	119
HL-60 - CD33KO	0%	36
A549	0%	LOD

Supplemental Table 2: Rapamycin half-life in NSG mice as a function of input dosing.

Supplemental Table 2

Dose Level (mg/kg)	Analysis Day	T_{max} (h)	T_{min} (h)	$T_{1/2}$ (h)	C_{max} (ng/mL)	C_{min} (ng/mL)	C_{48h} (ng/mL)	AUC-T_{0-48} (ng.h/mL)
0.02	1	2	48	16.0	13.7	1.16	1.16	226
0.05	1	1	48	17.7	44.9	3.68	3.68	666
0.1	1	2	48	13.8	118.0	5.49	5.49	1480
0.02	19	2	96	25.1	19.0	0.80	2.27	318
0.05	19	2	72	21.8	34.9	1.35	5.03	621
0.1	19	2	96	24.3	68.3	3.22	6.91	1020

Supplemental Table 3: Red Blood Cell Partitioning of rapamycin in NSG Mouse Whole Blood (K_2EDTA)

Supplemental Table 3

Compound	Conc. in Spiked Plasma / Conc. In Plasma from Spiked Blood	Ratio Adjusted for Hematocrit	$K_{p\ (RBC/PL)}$	% Bound to RBC	% Stability in Plasma
Rapamycin (2mM)	0.55	1.06	0.0628	5.5%	109.4%

Supplemental Table 4: Rapamycin (2mM) Protein Binding in CD-1 mice, NSG mice, and Human Plasma (K_2EDTA)

Supplemental Table 4

Species	% Protein Free (Mean ± SD)	Fu Unbound Fraction (Mean ± SD)	% Protein Bound (Mean ± SD)	% Stability in Matrix (2.5 hours)
CD-1 Mouse	0.07 ± 0.20	0.0007 ± 0.0002	99.93 ± 0.02	104.23
NSG Mouse (undiluted)	0.06 ± 0.30	0.000553 ± 0.000341	99.94 ± 0.03	191.90
Human	6.10 ± 8.20	0.061 ± 0.0082	93.90 ± 0.82	76.07

Supplemental Table 5: Rapamycin (2mM) Protein Binding in 10% NSG Mouse Plasma (K_2EDTA)

Supplemental Table 5

Species	% Free Measured in Diluted Plasma	% Free Estimated in Undiluted Plasma	Fu (unbound Fraction) Measured in Diluted Plasma	Fu (Unbound Fraction) Estimated in Undiluted Plasma	% Protein Bound Measured in Diluted Plasma	% Bound Estimated in Undiluted Plasma	% Stability in Matrix (2.5 Hours)
10% NSG Mouse	6.82 ± 1.04	0.73 ± .1.10	.0682 ± 0.0104	.00728 ± 0.00111	93.18 ± 1.04	99.27 ± 0.11	75.1
